# Supplementary material for: Pain Control by Co-adaptive Learning in a Brain-Machine Interface
Source: Curr Biol. 2020 Oct 19;30(20):3935–3944.e7. doi: 10.1016/j.cub.2020.07.066 (PMC7575198; doi:10.1016/j.cub.2020.07.066)
Supplement: Document S1. Figures S1–S4 and Table S1 [file mmc1.pdf]

Current Biology, Volume *30*

Supplemental Information

Pain Control by Co-adaptive Learning in a Brain-Machine Interface

Suyi Zhang, Wako Yoshida, Hiroaki Mano, Takufumi Yanagisawa, Flavia Mancini, Kazuhisa Shibata, Mitsuo Kawato, and Ben Seymour

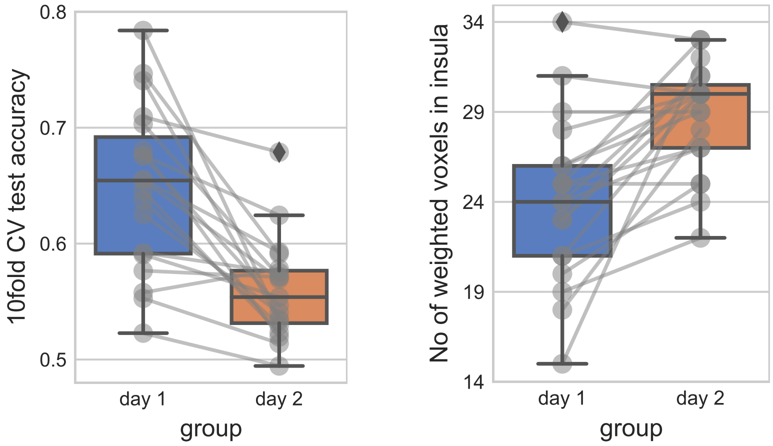

A B

Figure S1: **Decreased pain information in the insula. Related to Figure 3.**

(**A**) The 10 fold cross-validated test accuracy with bilateral insula ROI was 64.8% for day 1 and 56.0% for day 2 (Wilcoxon signed-rank test $Z(18)=3.69$, $p<0.001$).
(**B**) The decoder trained with day 2 data identified significantly more voxels as contributing to decoding performance compared to day 1 (day 1: 24.1±1.1 voxels, day 2: 28.7±0.7 voxels, signed-rank test $Z(18)=-3.21$, $p=0.001$). The sparse logistic regression method we used prunes unnecessary features/voxels when building the classifier [51]. There were no significant differences in the locations (i.e. average of x, y, or z coordinates) of these weighted voxels within individuals. Therefore both the searchlight and standard ROI decoder results suggest the insula cortex displays a disrupted pattern of information encoding for pain intensity on day 2.

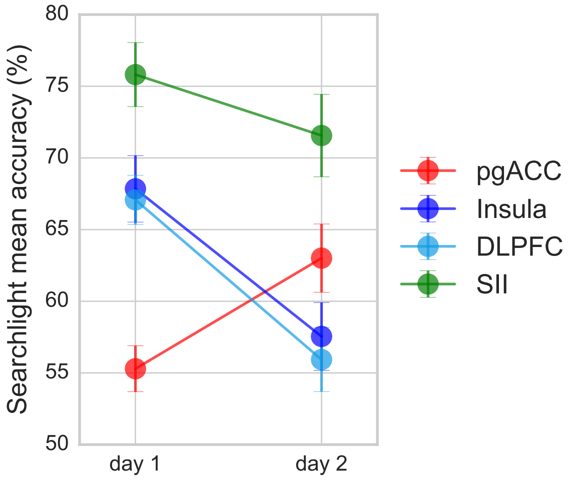

Figure S2: **Increased pain information in the pgACC. Related to Figure 3.**

Comparison between the mean searchlight accuracy map of pgACC and insula clusters (masks extracted from clusters shown in figures below, at $p<0.005$ unc.) and right SII (mask from accuracy>75% on day 1, k=50 voxels). The SII is known to robustly encode pain patterns and so could serve as an independent marker of changes, and its accuracy did not vary significantly across days (averaged within the ROI mask from day 1, k=50, day 1: 75.813 ± 2.234, day 2: 71.563 ± 2.880, paired t-test $T(18)=1.344$, $p=0.196$). A day$\times$location interaction showed a significant regional difference between pgACC and SII ($F(1,18)=12.1$, $p=0.0027$), although not significantly so between insula and SII ($F(1,18)=3.10$, $p=0.095$).

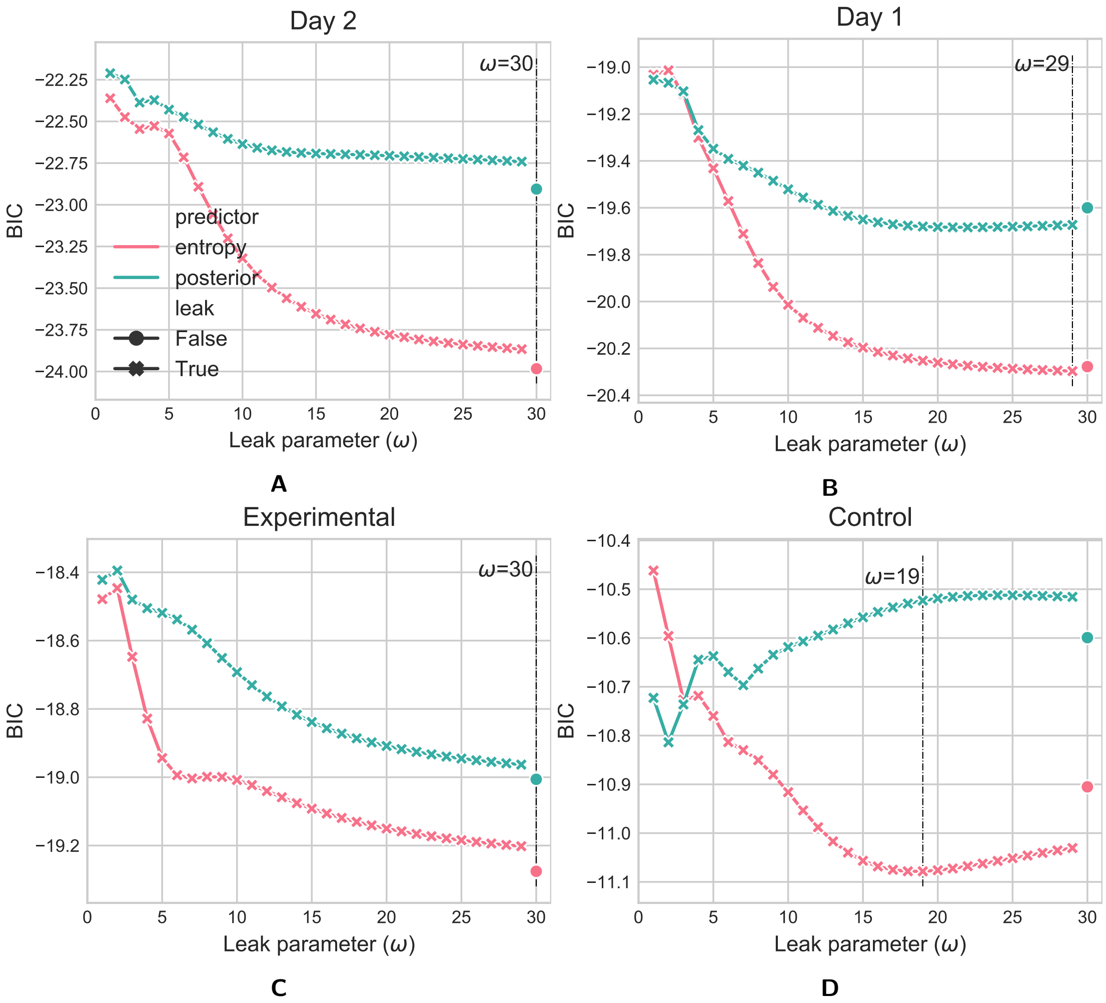

Figure S3: **Uncertainty model fitting and comparison. Related to Figure 4.**

(**A-B**) fMRI experiment: Model comparison showing entropy (formally representing uncertainty regarding upcoming stimulus level) from frequency learning model fitted pain rating residuals best (BIC the lower the better). Model with no leaky integration fitted day 2 data best (i.e. model assumed perfect tracking of trial frequency, day 1 BIC_{min}=-20.30, best-fit leaky parameter=29, day 2 BIC_{min}=-23.98, best-fit parameter: no leak). This suggests the model assumed perfect tracking of high/low pain frequency within a session of 30 trials on day 2, instead of exponentially weighted more recent trials on day 1, although the difference is close to negligible.
(**C-D**) EEG experiment: Model comparison showing entropy with no leak fitted pain rating residuals in the experimental group best (BIC_{min}=-19.28, best-fit parameter: no leak), however for the control group, the best-fit model included an integration parameter (ω = 19, BIC (BIC_{min}=-11.09) that discounts trials frequency earlier in the session.

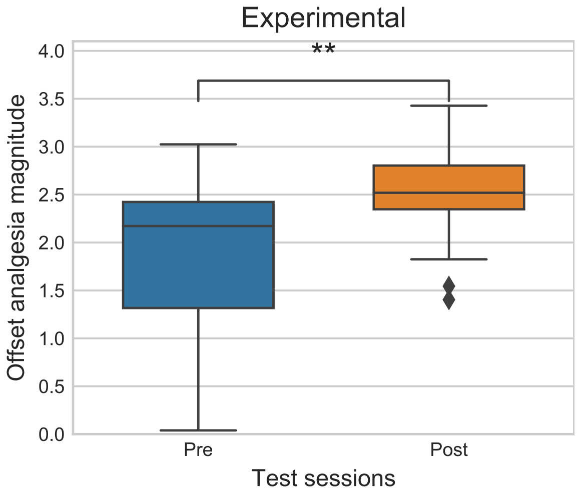

A

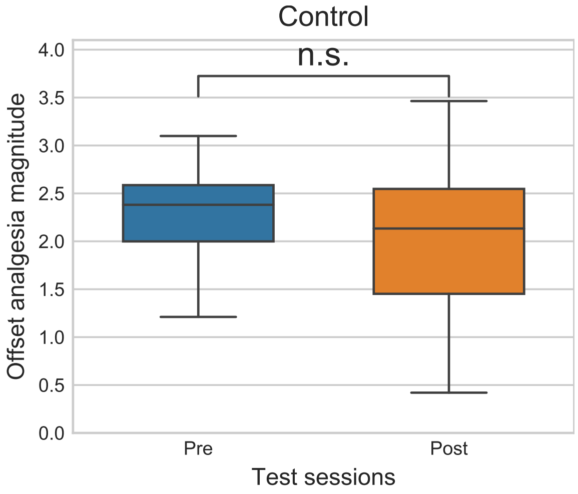

B

Figure S4: **Adaptive control increased temporal contrast enhancement. Related to Figure 5.**

The magnitude of temporal contrast enhancement before and after the task (pre/post) in the experimental (**A**) and control (**B**) groups. In the pre- and post- experimental temporal contrast enhancement task, subjects experienced a contact thermal pain stimulus that rose from a warm baseline to 45°C for 7 secs (T1), then to 46°C for 7 secs (T2), and then back to 45°C for 7 seconds (T3), and rated pain using a continuous numerical rating scale. Temporal contrast enhancement modulation magnitude is defined as pain ratings $T2_{max} - T3_{min}$.

Subject ID	Do you think the machine could read your pain and thus succeeded in reducing pain?	Did you tried to interact with the machine to exert some influence on it?	Actual strategies used (summarised)
1	Yes	Yes	Sometimes tried to prepare for the stimulus, sometimes simply wished for lower pain.
2	Yes	Yes	Concentrated on pain, several active strategies to change pain in the mind, including exaggerating response (i.e. make low pain lower, high pain higher)
3	Yes	Yes	No clear report of any cognitive strategy, generally passive
4	Yes	Yes	Attended to the pain, explicitly commented on intensity within his mind.
5	Yes	Yes	Thought about the pain, mainly wanting it not to hurt
6	Yes	Yes	Sometimes attended to the pain, including explicitly commented on intensity within his mind.
7	Yes	Yes	Concentrated on pain, sometimes trying to exaggerate the response
8	No	Yes	Mainly tried to relax
9	Yes	Yes	Concentrated on pain, some exaggeration (especially for high pain), related to pain to past memories.
10	Yes	Yes	Range of things: sometimes passive, sometimes attending to, exaggerating pain.
11	Yes	Yes	Sometimes passive, sometimes exaggerating pain
12	Yes	Yes	Attended to pain, monitored frequency, sometimes counting.
13	Yes	Yes	Generally passive
14	Yes	Yes	Focused on anticipating pain, sometimes tried to relate to pain memories
15	Yes	Yes	Tried to anticipate intensities, monitored frequency, often counting
16	Yes	Yes	Generally passive
17	Yes	Yes	Sometimes actively exaggerating pain, other times passive with mind wandering
18	Yes & No	Yes	Often exaggerated pain, other times more passive
19	Yes	Yes	Mainly focusing on pain, trying to decipher intensity clearly.

Table S1: **fMRI experiment post-training survey summary. Related to STAR Methods.**
