# Supplementary material for: Pain Control by Co-adaptive Learning in a Brain-Machine Interface
Source: Curr Biol. 2020 Oct 19;30(20):3935–3944.e7. doi: 10.1016/j.cub.2020.07.066 (PMC7575198; doi:10.1016/j.cub.2020.07.066)
Supplement: Document S2. Article plus Supplemental Information [file mmc2.pdf]

Current Biology

Article

Pain Control by Co-adaptive Learning in a Brain-Machine Interface

Graphical Abstract

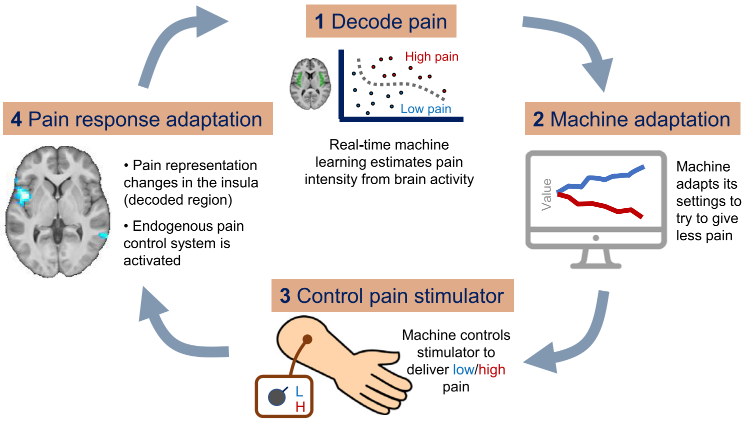

Authors

Suyi Zhang, Wako Yoshida, Hiroaki Mano, ..., Kazuhisa Shibata, Mitsuo Kawato, Ben Seymour

Correspondence

suyi.zhang@ndcn.ox.ac.uk (S.Z.),
kawato@atr.jp (M.K.),
ben.seymour@ndcn.ox.ac.uk (B.S.)

In Brief

Zhang et al. use real-time decoded fMRI responses from the insula cortex as input into a closed-loop control system aimed at reducing pain. They find that brain-machine interface control systems for pain induces a parallel set of co-adaptive changes in brain and behavior, illustrating the brain's ability to adapt to being decoded through learning.

Highlights

- Adaptive control system can use brain activity to guide interventions to reduce pain
- The brain changes when it knows it is being decoded
- These co-adaptive changes can disrupt decoded signals and modulate pain perception

Zhang et al., 2020, Current Biology *30*, 3935–3944
October 19, 2020 © 2020 The Authors. Published by Elsevier Inc.
https://doi.org/10.1016/j.cub.2020.07.066

CellPress

Current Biology

CellPress
OPEN ACCESS

Article

Pain Control by Co-adaptive Learning in a Brain-Machine Interface

Suyi Zhang,[1,2,6,7,*] Wako Yoshida,[2] Hiroaki Mano,[3] Takufumi Yanagisawa,[4] Flavia Mancini,[1] Kazuhisa Shibata,[5] Mitsuo Kawato,[2,*] and Ben Seymour[1,2,3,6,*]

[1]Computational and Biological Learning Laboratory, Department of Engineering, University of Cambridge, Cambridge, CB2 1PZ, UK

[2]Brain Information Communication Research Laboratory Group, Advanced Telecommunications Research Institute International, Kyoto 619-0237, Japan

[3]Center for Information and Neural Networks, National Institute for Information and Communications Technology, Osaka 565-0871, Japan

[4]Endowed Research Department of Clinical Neuroengineering, Global Center for Medical Engineering and Informatics, Osaka University, Osaka 565-0043, Japan

[5]Lab for Human Cognition and Learning, Center for Brain Science, RIKEN, Wako, Saitama 351-0198, Japan

[6]Wellcome Centre for Integrative Neuroimaging, University of Oxford, Oxford OX3 9DU, UK

[7]Lead Contact

*Correspondence: suyi.zhang@ndcn.ox.ac.uk (S.Z.), kawato@atr.jp (M.K.), ben.seymour@ndcn.ox.ac.uk (B.S.)

https://doi.org/10.1016/j.cub.2020.07.066

SUMMARY

Innovation in the field of brain-machine interfacing offers a new approach to managing human pain. In principle, it should be possible to use brain activity to directly control a therapeutic intervention in an interactive, closed-loop manner. But this raises the question as to whether the brain activity changes as a function of this interaction. Here, we used real-time decoded functional MRI responses from the insula cortex as input into a closed-loop control system aimed at reducing pain and looked for co-adaptive neural and behavioral changes. As subjects engaged in active cognitive strategies orientated toward the control system, such as trying to enhance their brain activity, pain encoding in the insula was paradoxically degraded. From a mechanistic perspective, we found that cognitive engagement was accompanied by activation of the endogenous pain modulation system, manifested by the attentional modulation of pain ratings and enhanced pain responses in pregenual anterior cingulate cortex and periaqueductal gray. Further behavioral evidence of endogenous modulation was confirmed in a second experiment using an EEG-based closed-loop system. Overall, the results show that implementing brain-machine control systems for pain induces a parallel set of co-adaptive changes in the brain, and this can interfere with the brain signals and behavior under control. More generally, this illustrates a fundamental challenge of brain decoding applications—that the brain inherently adapts to being decoded, especially as a result of cognitive processes related to learning and cooperation. Understanding the nature of these co-adaptive processes informs strategies to mitigate or exploit them.

INTRODUCTION

The management of human pain is in desperate need of innovation, given the magnitude of the clinical and societal problem and the limited success of conventional pharmacological treatments. Advances in machine-learning analysis of brain responses (‘brain decoding’) offer not just new insights into the neural representation of pain information [1], but they open up the possibility of using this information for novel biomedical technologies. In particular, real-time decoding of acute pain responses could in principle be used as a proxy biomarker to tune a therapeutic intervention—such as deep brain stimulation or spinal neuromodulation. By creating a closed-loop system, this allows the intervention to be constantly and automatically tracked and adjusted “online” to avoid over- or under-treatment [2–4]. However, closed-loop control is potentially most valuable when the intervention itself has multiple parameters, and whereby the optimal configuration and setting of these parameters is not known. The biomarker can then be used to guide algorithms to search and optimize them automatically—so-called *adaptive* control [5]. In this way, combining brain decoding with adaptive control algorithms can offer a powerful new approach to brain therapeutics.

Conventional approaches to decoding-based systems assume fixed, stable representations of the decoded state in the brain [6, 7]. However, this ignores the possibility of adaptive changes in the brain of the user, including cognitive processes such as intentionally trying to manipulate their brain activity for some purpose [8]. This is a general problem that affects many applications based on brain decoding, and the potential susceptibility of pain decoding-based biomarkers to cognitive modulation is recognized [9, 10]. It leads to the question of whether and to what extent a person can actively influence or control the decodability of information in their brain [11]. For instance, a user may want to enhance the clarity of their brain’s pain representation to make it easier for a putative therapeutic system to decode their pain and appropriately intervene on their behalf.

This is an open access article under the CC BY license (http://creativecommons.org/licenses/by/4.0/).

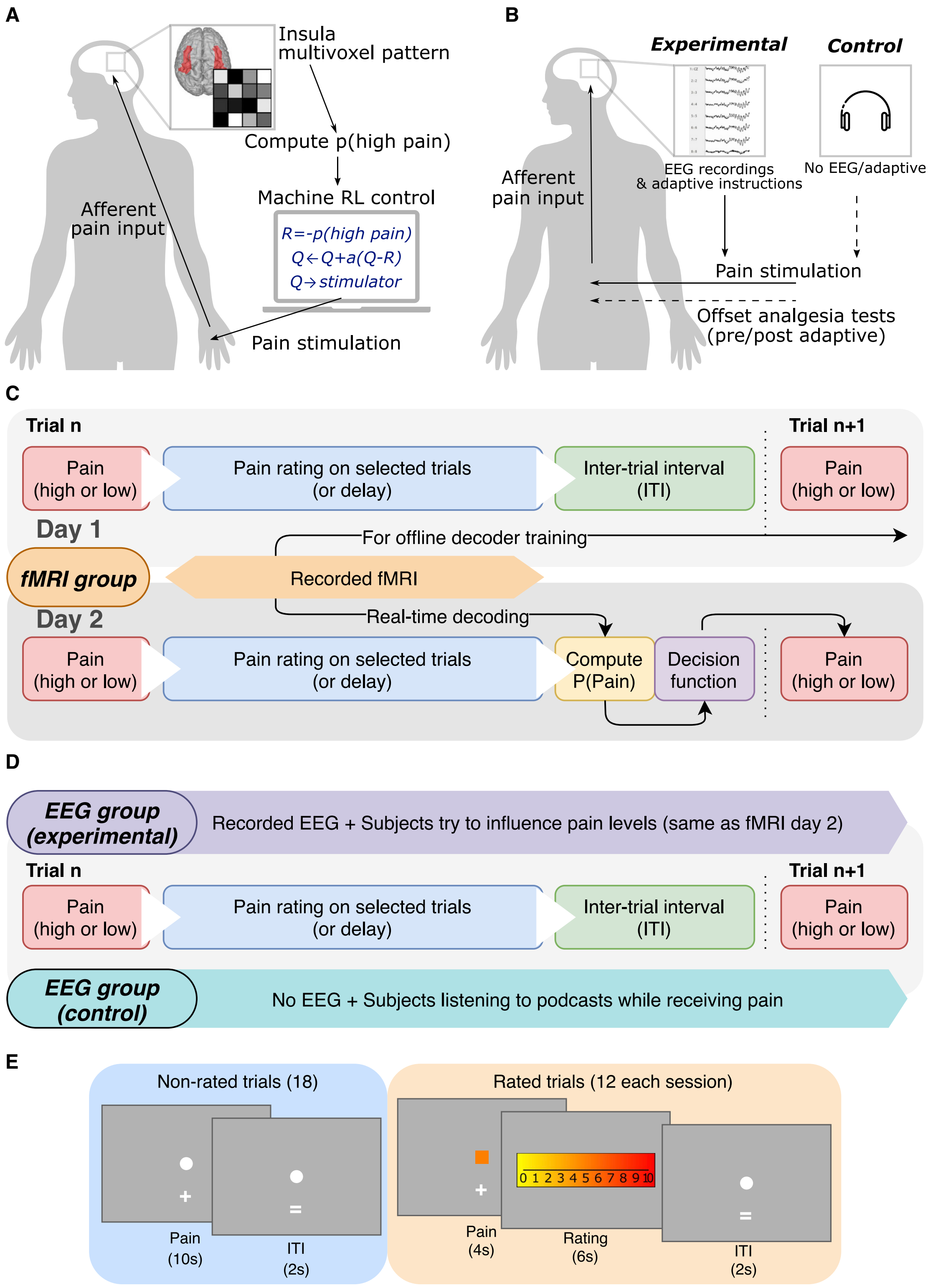

Figure 1. Experimental Paradigm

(A) Schematic illustration of the experimental setting for fMRI group in which the insula MVPA pain pattern is used to calculate feedback for an adaptive stimulus-control algorithm to learn which of two electrical stimulators was less painful to the subject.

(B) Illustration of EEG groups setting, in which experimental group had EEG recordings and the same instructions as fMRI group (day 2 adaptive control), while the control group received pain without EEG recordings or instructions (they just listened to audio-book that was not linked to the pain).

(legend continued on next page)

CellPress
OPEN ACCESS

Table 1. Insula Decoder Testing Performance

	Train and Test D1 (CV)	Train D1, Test D2	Train and Test D2 (CV)	Train D2, Test D1
Accuracy	0.649 (0.016)	0.563 (0.016)	0.560 (0.010)	0.491 (0.031)
Sensitivity	0.602 (0.026)	0.506 (0.016)	0.498 (0.031)	0.438 (0.026)
Specificity	0.665 (0.025)	0.631 (0.037)	0.590 (0.025)	0.549 (0.031)
# features (voxels)	24.05 (1.05)		28.74 (0.700)	

High pain = positive, low pain = negative for sensitivity/specificity calculation; CV, 10-fold cross validation; D1, day 1; D2, day 2. All values are mean (SEM), n = 19

This is potentially pernicious—most cognitive strategies to make pain clearer to decode from brain activity would involve paying attention to it. Attention enhances information processing in several ways, including augmenting learning toward external information that is uncertain, which allows future events (such as pain) to be more accurately predicted in the future [12, 13]. One key way in which learning is thought to be enhanced is by engaging the endogenous pain modulation system by modulating the descending pathways that control incoming nociceptive input in the spinal cord. For instance, facilitating pain increases its saliency and therefore enhances learned values and responses associated with it [14, 15]. Because of this, attention would ultimately be expected to alter the brain representation of pain and, in turn, would influence the accuracy of any *a priori* trained decoding-based biomarker. In other words, the cognitive process of trying to enhance a biomarker of pain in the brain might paradoxically disrupt it. This illustrates an important general point which arises when implementing adaptive brain-machine interfaces: do they induce parallel co-adaptive changes in the brain?

This study set out three goals. First, we aimed to establish whether, in principle, brain representations of pain can be decoded in real time from brain responses (functional MRI and EEG) and used to instruct an adaptive search algorithm linked to a pain-relief intervention; this would show in principle that adaptive control systems can be applied to pain. Second, we aimed to determine whether the neural representation of pain changes when subjects know the system is operational and have the opportunity to mentally control their brain activity. Third, we aimed to identify whether the endogenous pain modulation system is engaged by attention during the task, thus directly influencing the perception of pain.

RESULTS

Creating an Adaptive Control System Using Real-Time fMRI Decoding

We designed an fMRI-based closed-loop system using phasic, noxious stimuli. We aimed to train an adaptive control system to automatically learn how to reduce the intensity of stimulation based purely on decoding brain responses to preceding pain stimuli. This is essentially a bioengineering problem that needs to solve several core problems: training a voxel-wise pain classifier that can successfully generalize over time, re-positioning subjects with voxel-level accuracy in the fMRI scanner over days, implementing online classification using real-time fMRI, and using the output of such classification as input into a control algorithm to adjust subsequent stimulation.

To do this, we set up an experiment that took place over 2 days. The purpose of the first day ("decoder construction") was to allow us to build a decoder, using offline multivoxel-pattern analysis (MVPA), that could subsequently be used for online decoding in the adaptive control system the following day. On day 1, healthy subjects (19 total, 2 female) received a sequence of painful stimuli, delivered by either a high-intensity or low-intensity electrical stimulator, via a shared electrode attached to the left hand. The number of stimuli was roughly balanced between high and low pain, although not precisely given the fact that the order of stimuli on day 1 was actually yoked across subjects to the order delivered on day 2 (explained below). On day 1, subjects simply performed intermittent pain ratings, but other than that, there were no task demands. After the task, we used trial-based blood oxygen level-dependent (BOLD) responses from bilateral insula cortex to train the MVPA decoder to classify the two intensity levels. We chose the insula because it is known to have a primary role in pain encoding, and thus should be sufficient to support an adaptive control system [10, 16–19] (Figure 1A).

Returning on day 2 ("adaptive control"), the subjects experienced pain in a closed-loop adaptive control setting, with the basic principle being to use brain activity to control the pain stimulation. Specifically, after the subjects received a pain stimulus, we performed online classification of pain intensity using real-time fMRI BOLD signal from the insula, based on the offline decoding analysis from day 1. For each stimulus, the algorithm estimates the probability the intensity was high or low. This probability acted as the sole input to the control computer. The goal of the control computer was to figure out which of the two stimulators delivered the lower intensity pain and then preferentially trigger this stimulator. The online decoder therefore provided the feedback signal to allow it to work out which stimulator to trigger: in other words, a higher decoding accuracy would subsequently lead to lower pain.

At the beginning of each session, the control computer was naive to which electrical stimulator delivered high or low stimuli, and so it would choose either stimulator randomly. Based on a simple trial-and-error control algorithm (a reinforcement learning model), it used the decoder output as the feedback signal to learn a "value" term for each stimulator; the control computer then used the values assigned to each stimulator to determine which stimulator to trigger on the next trial. That is, a stimulator will acquire a high value if it is associated with a low classification probability of high pain, and this will lead to it being preferentially chosen.

(C) Trial structure for fMRI group on both days. fMRI images recorded on day 1 were used to train pain level decoders to be used on day 2, and real-time decoded information on day 2 were used by the stimulus reinforcement learning (RL) control system to decide on the pain level to deliver on the next trial.
(D) Similar trial structure was used for both EEG groups, with differences in EEG collection and instructions.
(E) Illustration of rated trials and timeline for fMRI group.

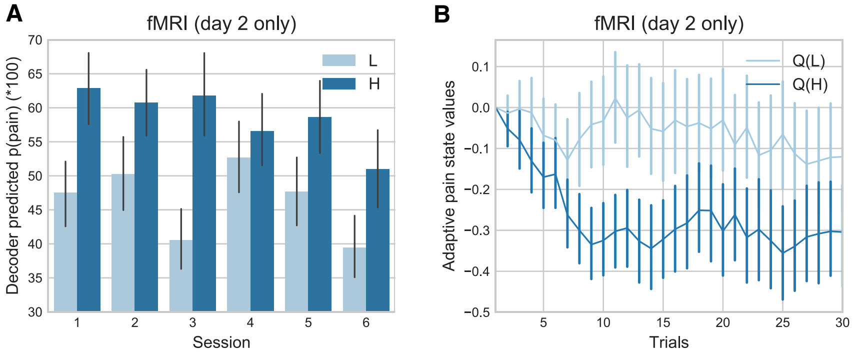

Figure 2. fMRI Behavioral Results

(A) Decoder predicted probabilities of having received high pain, P(pain), were able to distinguish high/low pain state (calculated for day 2 only).

(B) Within-session, the control system learned to value low pain states higher than high pain states (Q(L)>Q(H)) (day 2 only). H, high pain; L, low pain.

Mean ± SEM, n = 19 on each day.

Therefore, as long as the decoder from day 1 successfully generalizes to day 2, then the control algorithm should start to learn the values correctly. By adding some noise to the choice (stimulator selection) process, the control algorithm effectively samples each stimulator to build a reliable estimate of the value of each (''exploration''), which then allows it to trigger the low intensity stimulator most of the time (''exploitation'') (Figure 1C).

We fully explained the closed-loop set-up to the subjects so that they understood that (1) the control computer was trying to learn how to reduce their pain based on their brain activity, and (2) the control computer would be more able to give low pain if it could reliably ''read'' their pain signals. This therefore generated the incentive for subjects to enhance their brain responses to better communicate their pain signals. A post-experimental questionnaire confirmed that subjects both understood this, and most subjects actively engaged in various cognitive strategies to support this, such as focusing on the pain (see Table S1).

Decoder Classification Was Above Chance on Day 1

In terms of the success of the basic set-up, within-subject decoder construction based on the insula region of interest (ROI) achieved moderate classification accuracy, with a 10-fold cross-validated test accuracy of 65% (sensitivity 60%, specificity 67%, accuracy one-sample t test versus 0.5 across subjects: $T(18) = 8.967$, $p<1e{-7}$), shown in Table 1.

Decoder Classification Generalized to Day 2 (Adaptive Control)

When this classifier was used on day 2 for adaptive control, real-time decoding accuracy remained above chance, indicating successful generalization of the decoder across days (day 2: accuracy 56%, sensitivity 51%, specificity 63%, accuracy t test versus 0.5: $T(18) = 4.053$, $p = 0.0007$). Specifically, the real-time decoder classification of high pain (referred to as P(pain), Figure 2A) was significantly greater after delivery of a true high-pain stimulus compared with a low-pain stimulus (repeated-measure ANOVA of session and pain level effects, only pain level main effect significant: $F(1,18) = 17.41$, $p = 0.0006$, bootstrapped 95% CI P(pain) for high pain = [0.545, 0.660], low pain = [0.410, 0.524]).

Decoded Signals Allowed the Adaptive Control System to Preferentially Deliver Low Pain

Decoder performance was therefore sufficient for the control algorithm to learn differential decision values for high and low pain stimulators within a few trials in each new session (Figure 2B, mean ± SEM in arbitrary units of value, high pain = −0.264 ± 0.0486, low pain = −0.0608 ± 0.0479, paired t test: $T(18) = -3.651$, $p = 0.0018$). Given these differential values, the control system was able to deliver significantly fewer high compared with low pain stimuli (fMRI day 2 high-pain percentage: 43.480% ± 2.353%, one-sample t test versus 50%: $T(18) = -2.771$, $p = 0.0126$). Therefore, the control algorithm successfully learned to reduce pain. This achieved the first experimental goal, showing that it is possible in principle to design an adaptive control system for pain based on brain activity.

Changes in Pain Representations during Adaptive Control

To identify potential brain-wide changes in pain representations during adaptive control, we used a whole-brain post hoc MVPA searchlight analysis. This effectively performs a decoding analysis independently on each day within a roaming ROI and evaluates the contribution each voxel makes to classification accuracy within each day. This analysis measures the pain information content in each voxel. For instance, although the day 1 decoder doesn't perform as well on day 2 versus day 1, this doesn't in itself mean that the insula information content is reduced because the other factors alone may achieve this, such as slight decoder over-fitting and small errors in subject re-positioning. However, since the searchlight analysis considers classification performance *within* each day, we can get an independent, brain-wide accuracy map for each day. And by comparing day 2 to day 1 (paired t test, DF = 18), we can calculate an accuracy map that reflects a *change* in information content during adaptive control [1, 20].

Decreased Pain Information in the Insula

We found reduced pain-level decoding accuracy localized to a region in the left mid/anterior insula (Figure 3A; Table 2; [−45, 6, 2]; $T = -6.04$, $k = 142$, effect size Cohen's $d = -1.386$, whole-brain cluster level p(FWEcorr) = 0.014). Extracting the exact values from accuracy maps from both days, decoder classification performance (percentage) reduced from 67.844 ± 2.320 on day 1 to 57.546 ± 2.366 on day 2 (171 voxels, paired t test $T(18) = -5.335$, $p = 4.525e{-5}$) in the left insula (Figure 3A, see Supplemental Information for additional analyses). This shows that the reduced decoder performance during adaptive control on day 2 must be more than what can be explained by generalization factors and represents a significant reduction in pain information content itself. Outside of our insula ROI, we did not see decreased information content anywhere else in the brain at corrected thresholds. Even at a liberal uncorrected threshold, only the left middle frontal gyrus displayed a possible reduction (see Table 2).

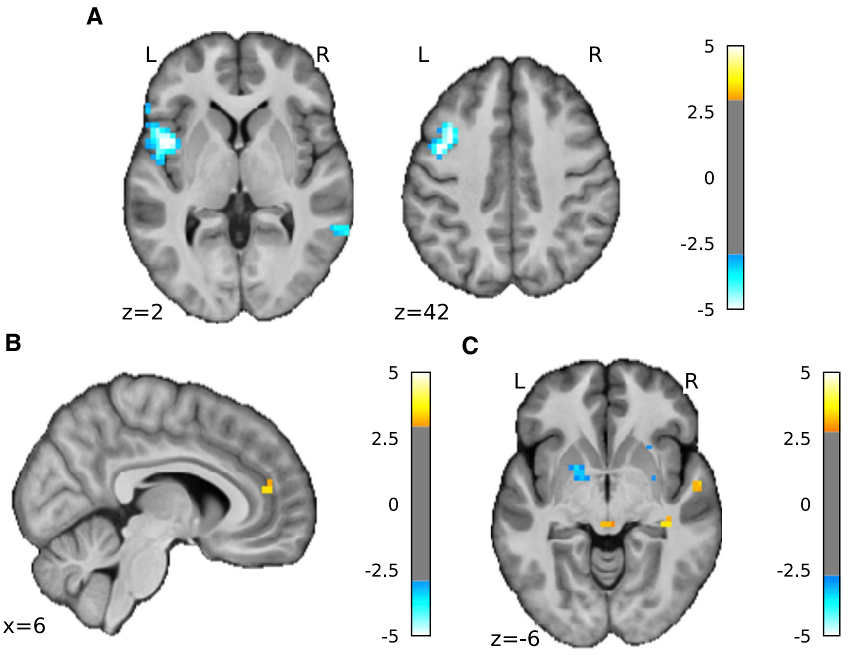

Figure 3. fMRI Searchlight Analysis Results

(A) Searchlight analysis showed that information content contributing to decoding accuracy decreased in left insula on day 2 compared with day 1 (shown at $p < 0.001$, k>0 for display purposes, see Table 2 for statistics).

(B) Information content contributing to decoding accuracy increased in pgACC day 2 > day 1 (shown at $p < 0.005$, k>0 for display purposes, see Table 2 for statistics).

(C) Univariate whole-brain comparison (2nd level paired t test, day 2>day 1) of the high pain>low pain first level contrasts, interaction were observed in the PAG (peak coordinates [0, −30, −6], T = 3.27, k = 3) (shown at $p < 0.005$, k>0 for display purposes, see Table 2 for statistics).

Mean ± SEM, fMRI group n = 19.

See also Figures S1, S2.

Increased Pain Information in the pgACC

In contrast, we found that information content was *increased* in the pregenual anterior cingulate cortex (pgACC) (Figure 3B shown at $p < 0.005$ uncorrected; Table 2; [6, 44, 14]; T = 3.50, k = 5, Cohen's d = 0.803, small volume correction (SVC) using an 8-mm spherical mask based on our previous investigation [14]). The pgACC was a target region of interest because we have shown that it has a specific role in endogenous modulation and cognitive control in adaptive settings (alongside the periaqueductal gray (PAG) [21]). Extracting the exact values from the accuracy maps from both days, the pgACC ROI had significantly increased decoding accuracy across all participants (Figure S2, day 1 accuracy: 55.293 ± 1.604, day 2: 63.009 ± 2.383, paired t test T(18) = 3.676, p = 0.0017). No other brain regions were identified as showing an increase in decoder accuracy, even at a liberal exploratory threshold.

In summary, we found evidence in support of our second hypothesis that pain representations were altered in the brain; crucially, pain encoding in the insula—a primary pain processing region—was disrupted, while information encoding was enhanced in the pgACC.

Evidence of Endogenous Modulation during Adaptive Control

Our third main hypothesis was the prediction that subjects' cognitive engagement with adaptive control enhances endogenous modulation of pain. Although the increased pain information in pgACC reported above would be consistent with this, further analysis of brain and behavioral responses is needed to provide more robust evidence.

Increased PAG Univariate Responses

We first looked at univariate differences in brain activity to identify any straightforward increase in brain responses, especially in the PAG. The PAG is the primary mediator of descending control that relays cortical messages to the dorsal horn of the spinal cord and receives projections from the pgACC [22]. Whole-brain analysis of fMRI data using a conventional general linear model showed evidence of a regional day × pain level interaction in the PAG (Figure 3C shown at $p < 0.005$ uncorrected). Specifically, within-subject comparison (day 2>day 1) of the contrast (high pain>low pain) confirmed increased responses in the PAG (peak coordinates [0, −30, −6], T = 3.27, k = 3, Cohen's d = 0.750, p = 0.048 after small volume correction for multiple comparisons), but in no other regions. This provides additional neural evidence that the endogenous control system is more active on day 2 during adaptive control.

Uncertainty Correlated with Subjective Pain Rating

In line with the hypothesis that an attentional mechanism underlies engagement of the endogenous control system, we looked for evidence that pain ratings were correlated with uncertainty during adaptive control. The primary learnable information in the task is the relative frequency of high and low pain, as this indicates how well the adaptive control system is working. On a trial-by-trial basis, the uncertainty measure quantifies how much new information is available and directs attentional resources to enhance learning accordingly [12]. Therefore, any correlation of uncertainty with pain ratings would be consistent with attention-related endogenous modulation. Using a standard model of frequency learning [23, 24], we found that the uncertainty was indeed significantly positively correlated with pain ratings on day 2 (adaptive control) but not day 1 (decoder construction) (z-transformed correlation coefficients day 2: 0.172 ± 0.039, t test versus 0: T(18) = 4.356, p = 3.81e-4, day 1: 0.0090 ± 0.052, T(18) = 0.944, p = 0.358, a direct day 2 versus day 1 contrast was not significant).

Uncertainty Correlated with pgACC Activity

We therefore studied the brain imaging data to see whether uncertainty also correlated with brain responses—especially in the pgACC, the putative control center for attentional endogenous control [15]. We found that uncertainty was indeed positively correlated with BOLD responses in the pgACC (Figure 4A) in a location that overlapped with the region associated with enhanced decoding accuracy during adaptive control

Table 2. Experiment 1 Multiple-Comparison Correction

p*	k	T	Z	MNI Coordinates (mm) x	y	z	Region Mask
Figure 3. Searchlight Analysis—Decreased Information Content (D2>D1)							
0.048	2	3.94	3.3	−42	3	−2	Insula L
0.061	2	4.41	3.59	−38	15	42	Middle frontal gyrus L
0.078	1	4.37	3.56	−38	35	30	
Figure 3. Searchlight Analysis—Increased Information Content (D2>D1, display at p < 0.005)							
0.045	5	3.50	3.02	6	44	14	8 mm pgACC sphere at [6, 40, 12] [14]
Figure 3. Whole-Brain Comparison (D2>D1, H>L, display at p < 0.005)							
0.048	1	3.23	2.83	−3	−30	−6	PAG [46]
Figure 4. Frequency Learning Model—Posterior Probability of Low Pain (D2)							
0.007	10	4.44	3.6	0	51	−14	Frontal medial cortex
Figure 4. Frequency Learning Model—Entropy (D2)							
0.039	5	5.30	4.06	10	41	10	Cingulate anterior
0.033	6	4.36	3.56	0	3	38	
0.002	14	5.91	4.35	13	41	14	8 mm pgACC sphere at [6, 40, 12] [14]
0.002	31	5.24	4.03	−38	−7	2	Insular cortex (bilateral)
0.032	6	4.60	3.69	39	−4	6	

cluster-forming threshold of p <0.001 uncorrected unless stated otherwise. Small-volume correction performed with ROI masks from Harvard-Oxford, PAG probabilistic atlas, and previous study. *FWE cluster-level p value. n = 19. H, high pain; L, low pain.

(Figure 4B). When comparing with day 1, we found that the peak pgACC response was significantly greater on day 2 (SVC corrected p(FWE-corr) = 0.021, T = 3.70, Z = 3.15, peak coordinates [13, 41, 14], Cohen's d = 0.849). That is, uncertainty correlated with both pain ratings and pgACC BOLD responses during adaptive control (i.e., day 2).

In summary, both behavioral and neural evidence indicated engagement of the endogenous modulatory system during adaptive control, suggesting that subjects' active strategies in engaging with the adaptive control system drove an attention-like modulation of pain responses that was evident in pgACC.

Experiment 2: Further Evidence that Adaptive Control Engages the Endogenous Modulation System

To provide more robust behavioral evidence of the engagement of the endogenous modulation system, we performed a second, EEG-based experiment in which we evaluated endogenous control in a temporal contrast enhancement task before and after the adaptive control task (Figures 1B and 1D; see STAR Methods). Temporal contrast enhancement describes the well-known phenomenon in which small increases or decreases in tonic pain induce an exaggerated effect on pain ratings ("onset hyperalgesia" and "offset hypoalgesia" [25–27]). This "hypersensitivity to change" is known to involve descending facilitation and suppression and is likely to be mechanistically related to attentional modulation because it reflects the importance of sudden changes in pain as a driver of attention and learning [15].

Uncertainty Correlated with Subjective Pain Rating

First, during the adaptive control task itself, we again applied the frequency learning model as in the fMRI experiment to look for a correlation between pain ratings and uncertainty. We found a positive correlation between the uncertainty model and pain ratings in the experimental group, similar to, albeit slightly weaker than, the fMRI group, and no correlation in a control group in which subjects received pain outside of the context of adaptive control (z-transformed correlation coefficients experimental versus 0: T(27) = 2.115, p = 0.0438, control versus 0: T(27) = 1.304, p = 0.203).

Adaptive Control Increased Temporal Contrast Enhancement

In the pre- and post-experimental temporal contrast enhancement task, subjects experienced a contact thermal pain stimulus that rose from a warm baseline to 45°C for 7 s (T1), then to 46°C for 7 s (T2), and then back to 45°C for 7 s (T3), and rated pain using a continuous numerical rating scale. Figures 5A and 5B show the normalized modulation of pain rating traces before and after the task (pre/post) in the experimental and control groups, respectively. Modulation magnitudes were significantly positive for the experimental group (0.0531 ± 0.025, T(27) = −3.109, p = 0.0044) but not control (0.0339 ± 0.026, T(27) = 1.446, p = 0.160), with a significant group × pain level interaction (repeated-measure ANOVA F(1,54) = 11.443, p = 0.0013). Specifically, comparing post->pre-magnitude (the absolute difference between the maximal pain rating in T2 and the minimum in T3) across groups, an effect size of 0.904 was observed (experimental: 0.658 ± 1.120, control: −0.209 ± 0.764, Cohen's d bootstrapped 95% CI [0.444, 1.392], repeated-measure ANOVA task × group interaction F(1,54) = 11.538, p = 0.0013; see Figure S5). In summary, the data from this experiment showed that adaptive control enhances a behavioral measure of endogenous modulation of pain, both during and after adaptive control.

DISCUSSION

The experiments addressed our three questions. First, we showed that the brain representation of pain can be decoded in real-time to build an adaptive control system. Even with only moderate decoding accuracy, this system can learn to find an

CellPress
OPEN ACCESS

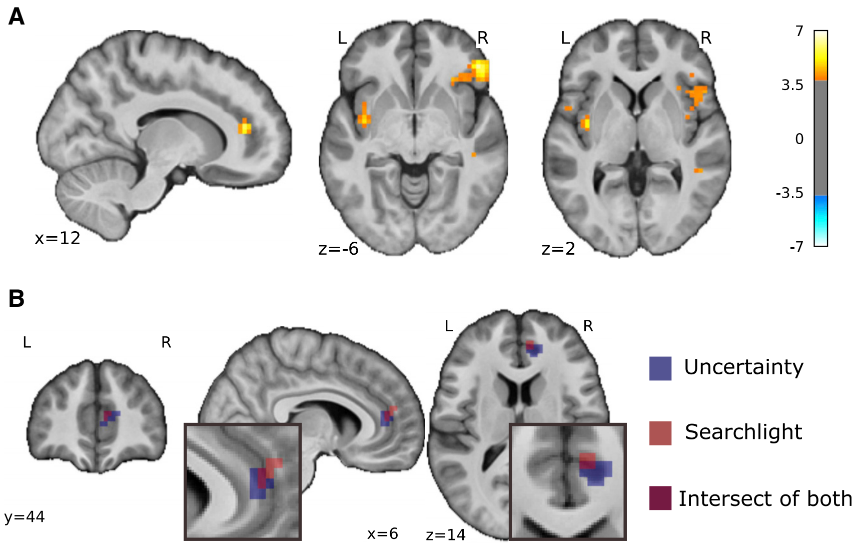

Figure 4. Frequency Learning Model Neural Correlates

(A) Uncertainty on fMRI day 2 (i.e., entropy of posterior probability of current stimulus before updating) correlated with pgACC and bilateral insula (pgACC peak coordinates [13, 41, 14], T = 5.91, Cohen's d = 1.36, sagittal and coronal views both at $p < 0.001$ uncorrected, see Table 2 for multiple correction statistics).

(B) Overlay of pgACC activation from both uncertainty (blue) and searchlight (red) analysis (uncertainty visualized at $Z>3.2$, searchlight at $Z>2.8$).

See also Figure S3.

intervention that reduces pain. Second, we showed the neural representation of pain changes under such a system, in parallel with the inherent engagement of learning and cognitive control. In particular, pain encoding in the insula is selectively disrupted, reducing the efficacy of this region to act as a biomarker to support control. Third, we showed this change in representation is associated with attention-related endogenous pain modulation, which in itself influences perceived pain. This is apparent both during adaptive control as a function of learning and afterward in conventional tests of endogenous pain modulation (temporal contrast enhancement). Overall, the study shows that implementing an adaptive control system for pain is technically feasible but that it induces a set of specific, co-adaptive changes in the brain.

From a clinical perspective, closed-loop systems that use brain-based biomarkers have been advanced for deep brain stimulation for Parkinson's disease and epilepsy, where clear disease-specific biomarkers are well established [28–30]. Clinical pain is known to display substantial temporal fluctuations and drifts [31], and so it should be much more efficient to use an "automated" brain-based system to tune a putative intervention, as opposed to using continual self-report (the gold standard for pain measurement). However, rather than using a "hardwired" control system in which the appropriate intervention is known and thus fixed in advance, here, we introduce an adaptive control system that learns from experience. This is potentially powerful because, for many applications, the best intervention (such as the configuration for amplitudes and frequency of a multi-electrode deep-brain or spinal stimulator) is not known in advance. Using an adaptive framework based on reinforcement learning offers enormous potential advantages given its ability to learn high-dimensional problems, reuse system knowledge for efficiency, and incorporate human prior knowledge within the control architecture [32, 33].

The development of sophisticated control systems inevitably benefits from more accurate biomarkers. While multi-region/brain-wide biomarkers for phasic pain can exceed 90% accuracy [7], a single-region biomarker may be more relevant to clinically applicable brain recording systems (such as implantable systems [34]). Utilizable systems would also ideally decode pain rating directly—that is, using a multivariate regression over ratings, instead of a high/low classification. However, a greater concern is that the potential accuracy of single-region biomarkers for clinical chronic pain, as opposed to experimental phasic pain, remains unclear, and this represents one of the biggest hurdles to any clinical realization of adaptive control systems.

There are several reasons why the fidelity of biomarker decoding for a brain-machine interface may change with time, including various technical or hardware issues. However, the induction of co-adaptive learning and cognitive changes has received little attention. Any control system that uses brain activity in principle generates the incentive for the subject to try and voluntarily modulate their brain activity to influence the signals being read and interpreted. Increasing the neural discriminability of pain is different from common notions of cognitive pain control, such as overall pain suppression. Indeed, it is not clear exactly what one should do, in terms of a cognitive strategy, to enhance brain-machine communication in this respect. However, based on the post-training survey, most subjects engaged in some form of active strategy, and this typically involves an increase in attention to pain, for instance, as they think about how well the machine is reading their pain. From a practical perspective, understanding the brain's co-adaptive strategies to being decoded may be a first step toward assessing the long-term impact of such control systems, which also informs their design and associated risks.

This leads to the question of why such attention to pain did not result in an increased discriminability of pain intensity in the insula. One possible explanation is that the representation of pain intensity was disrupted by the co-representation of uncertainty that arose as a function of learning the distribution of pain intensities (i.e., the relative frequency of high and low pain). That is, the insula may be encoding more than simply pain intensity [19], and this limits generalizability of a decoder when the cognitive context changes in a way that captures the other variables that the insula encodes. The best way around this problem in the future would be to intermittently retrain the decoder, ideally in the context of an operating adaptive control system.

The change in pain representation seen in the insula raises the issue of what happens to the subjective perception of pain when

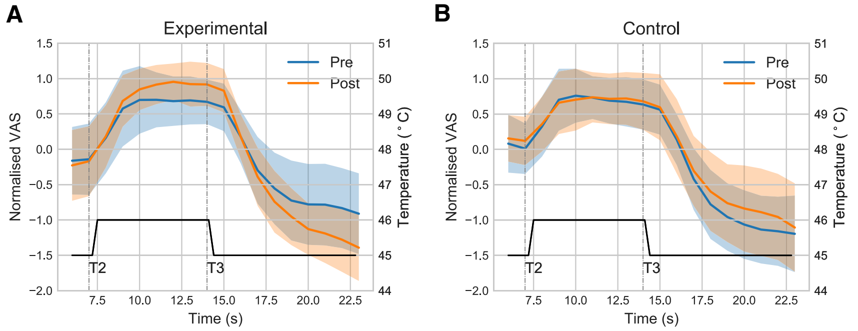

Figure 5. EEG Behavioral Results
Temporal contrast enhancement task showed pain rating traces when comparing pre-/post-adaptive control sessions, exaggerated pain and pain-relief responses were observed in (A) the experimental group only, as compared with (B) the control group (shaded regions are standard deviation). n = 28 each in experimental and control group.
See also Figure S4.

people engage with a brain-machine interface that implements adaptive control. From a psychological perspective, we proposed that cognitive engagement would often involve increased attention to pain, as subjects either attempt to manipulate how they perceive pain or simply monitor or evaluate the effectiveness of the system. Because attention itself modulates pain to drive learning, we predicted neural and behavioral evidence of activation of the endogenous modulatory system should be observable. In the brain, this was manifested in the pgACC by higher discriminability of pain intensity and by the representation of uncertainty during learning. The pgACC is well recognized as a cortical control site for descending control on the basis of attention and cognitive controllability [35–42]. Engagement of endogenous control was also manifested in enhanced responses in the PAG, the primary descending control hub mediating projections to the spinal cord. Overall, these findings provide good neural evidence for enhanced endogenous modulation of pain during adaptive control.

Behaviorally, involvement of the endogenous control system predicts a specific effect on perceived pain. During adaptive control, this was manifested in terms of a positive correlation between pain and uncertainty. Uncertainty is presumed to increase pain to drive learning [43–45], and this was observed in both experiments, in keeping with simple models of frequency learning as subjects monitored the balance of high and low pain stimuli delivered by the machine. However, the impact of enhancement of endogenous control was also robustly seen in temporal contrast enhancement (onset hyperalgesia and offset analgesia) *after* the adaptive control. This implies a persistent and specific adaptive change in the endogenous control system.

In summary, this study shows that it is possible to design adaptive control systems that use brain activity to search for an intervention that reduces pain. However, it also shows that the brain does not sit passively when this is implemented. Instead, the brain changes when it knows it is being decoded. Specifically, a set of co-adaptive changes are induced that can both disrupt the signals used by the adaptive control system and modulate the perception of pain itself. This shows in principle that the design of any adaptive brain-machine interface needs to consider the co-adaptive changes that its implementation may induce.

STAR★METHODS

Detailed methods are provided in the online version of this paper and include the following:

- KEY RESOURCES TABLE
- RESOURCE AVAILABILITY
  - Lead contact
  - Materials availability
  - Data and code availability
- EXPERIMENTAL MODEL AND SUBJECT DETAILS
  - Participants
- METHOD DETAILS
  - Experiment 1: fMRI-based closed-loop control
  - Experiment 2: EEG-based closed-loop control

SUPPLEMENTAL INFORMATION

Supplemental Information can be found online at https://doi.org/10.1016/j.cub.2020.07.066.

ACKNOWLEDGMENTS

Research was supported by the "Application of DecNef for development of diagnostic and cure system for mental disorders and construction of clinical application bases" of the Strategic Research Program for Brain Sciences from Japan Agency for Medical Research and Development (AMED, JP17dm0107044, JP18dm0307008), the Wellcome Trust (097490), Arthritis Research UK (Versus Arthritis: 21357, 21192), the National Institute for Information and Communications Technology (NICT, Japan), the Institute of Information & Communications Technology Planning & Evaluation (IITP) grant funded by the Korea government (MSIT) (No. 2019-0-01371, Development of brain-inspired AI with human-like intelligence), and the Japanese Society for the Promotion of Science (JSPS); S.Z. was supported by the W.D. Armstrong Fund and the Cambridge Trust. K.S. was supported by JSPS Kakenhi (17H04789, 19H01041). F.M. was supported by Medical Research Council (MRC, MR/T010614/1). We thank the imaging teams at the Advanced Telecommunications Research Institute for their assistance in performing the study. We also thank Dr. Christian Sprenger for constructive comments on the manuscript.

AUTHOR CONTRIBUTIONS

S.Z. contributed to conceptualization, design, data acquisition, analysis, interpretation, and manuscript draft and editing. W.Y. contributed to design, data acquisition, interpretation, and manuscript draft and editing. H.M. contributed to data acquisition and manuscript draft and editing. T.Y., K.S., and M.K. contributed to data interpretation and manuscript draft and editing. F.M. contributed to the design and data acquisition of the EEG study and manuscript draft and editing. B.S. contributed to conceptualization, design, data acquisition, analysis, interpretation, funding acquisition, supervision, and manuscript draft and editing.

DECLARATION OF INTERESTS

The authors declare no competing interests.

CellPress
OPEN ACCESS

Received: April 16, 2020
Revised: June 22, 2020
Accepted: July 21, 2020
Published: August 13, 2020

REFERENCES

1. Kriegeskorte, N., Goebel, R., and Bandettini, P. (2006). Information-based functional brain mapping. Proc. Natl. Acad. Sci. USA *103*, 3863–3868.
2. Stanslaski, S., Afshar, P., Cong, P., Giftakis, J., Stypulkowski, P., Carlson, D., Linde, D., Ullestad, D., Avestruz, A.-T., and Denison, T. (2012). Design and validation of a fully implantable, chronic, closed-loop neuromodulation device with concurrent sensing and stimulation. IEEE Trans. Neural Syst. Rehabil. Eng. *20*, 410–421.
3. Zhang, S., and Seymour, B. (2014). Technology for chronic pain. Curr. Biol. *24*, R930–R935.
4. Shirvalkar, P., Veuthey, T.L., Dawes, H.E., and Chang, E.F. (2018). Closed-Loop Deep Brain Stimulation for Refractory Chronic Pain. Front. Comput. Neurosci. *12*, 18.
5. DiGiovanna, J., Mahmoudi, B., Fortes, J., Principe, J.C., and Sanchez, J.C. (2009). Coadaptive brain-machine interface via reinforcement learning. IEEE Trans. Biomed. Eng. *56*, 54–64.
6. Marquand, A., Howard, M., Brammer, M., Chu, C., Coen, S., and Mourão-Miranda, J. (2010). Quantitative prediction of subjective pain intensity from whole-brain fMRI data using Gaussian processes. Neuroimage *49*, 2178–2189.
7. Wager, T.D., Atlas, L.Y., Lindquist, M.A., Roy, M., Woo, C.-W., and Kross, E. (2013). An fMRI-based neurologic signature of physical pain. N. Engl. J. Med. *368*, 1388–1397.
8. Woo, C.-W., Chang, L.J., Lindquist, M.A., and Wager, T.D. (2017). Building better biomarkers: brain models in translational neuroimaging. Nat. Neurosci. *20*, 365–377.
9. Woo, C.-W., Roy, M., Buhle, J.T., and Wager, T.D. (2015). Distinct brain systems mediate the effects of nociceptive input and self-regulation on pain. PLoS Biol. *13*, e1002036.
10. Woo, C.-W., Schmidt, L., Krishnan, A., Jepma, M., Roy, M., Lindquist, M.A., Atlas, L.Y., and Wager, T.D. (2017). Quantifying cerebral contributions to pain beyond nociception. Nat. Commun. *8*, 14211.
11. Shibata, K., Watanabe, T., Sasaki, Y., and Kawato, M. (2011). Perceptual learning incepted by decoded fMRI neurofeedback without stimulus presentation. Science *334*, 1413–1415.
12. Dayan, P., Kakade, S., and Montague, P.R. (2000). Learning and selective attention. Nat. Neurosci. *3* (*Suppl*), 1218–1223.
13. Behrens, T.E., Woolrich, M.W., Walton, M.E., and Rushworth, M.F. (2007). Learning the value of information in an uncertain world. Nat. Neurosci. *10*, 1214–1221.
14. Zhang, S., Mano, H., Lee, M., Yoshida, W., Kawato, M., Robbins, T.W., and Seymour, B. (2018). The control of tonic pain by active relief learning. eLife *7*, e31949.
15. Seymour, B. (2019). Pain: A Precision Signal for Reinforcement Learning and Control. Neuron *101*, 1029–1041.
16. Brodersen, K.H., Wiech, K., Lomakina, E.I., Lin, C.S., Buhmann, J.M., Bingel, U., Ploner, M., Stephan, K.E., and Tracey, I. (2012). Decoding the perception of pain from fMRI using multivariate pattern analysis. Neuroimage *63*, 1162–1170.
17. Craig, A.D. (2002). How do you feel? Interoception: the sense of the physiological condition of the body. Nat. Rev. Neurosci. *3*, 655–666.
18. Segerdahl, A.R., Mezue, M., Okell, T.W., Farrar, J.T., and Tracey, I. (2015). The dorsal posterior insula subserves a fundamental role in human pain. Nat. Neurosci. *18*, 499–500.
19. Geuter, S., Boll, S., Eippert, F., and Buchel, C. (2017). Functional dissociation of stimulus intensity encoding and predictive coding of pain in the insula. eLife *6*, e24770.
20. Hebart, M.N., Görgen, K., and Haynes, J.-D. (2015). The Decoding Toolbox (TDT): a versatile software package for multivariate analyses of functional imaging data. Front. Neuroinform. *8*, 88.
21. Roy, M., Shohamy, D., Daw, N., Jepma, M., Wimmer, G.E., and Wager, T.D. (2014). Representation of aversive prediction errors in the human periaqueductal gray. Nat. Neurosci. *17*, 1607–1612.
22. Basbaum, A.I., and Fields, H.L. (1984). Endogenous pain control systems: brainstem spinal pathways and endorphin circuitry. Annu. Rev. Neurosci. *7*, 309–338.
23. Meyniel, F., Maheu, M., and Dehaene, S. (2016). Human Inferences about Sequences: A Minimal Transition Probability Model. PLoS Comput Biol. *12*, e1005260.
24. Mars, R.B., Debener, S., Gladwin, T.E., Harrison, L.M., Haggard, P., Rothwell, J.C., and Bestmann, S. (2008). Trial-by-trial fluctuations in the event-related electroencephalogram reflect dynamic changes in the degree of surprise. J. Neurosci. *28*, 12539–12545.
25. Yelle, M.D., Rogers, J.M., and Coghill, R.C. (2008). Offset analgesia: a temporal contrast mechanism for nociceptive information. Pain *134*, 174–186.
26. Szikszay, T.M., Adamczyk, W.M., and Luedtke, K. (2019). The Magnitude of Offset Analgesia as a Measure of Endogenous Pain Modulation in Healthy Subjects and Patients with Chronic Pain– A Systematic Review and Meta-analysis. Clin J Pain. *35*, 189–204.
27. Sprenger, C., Stenmans, P., Tinnermann, A., and Buchel, C. (2018). Evidence for a spinal involvement in temporal pain contrast enhancement. Neuroimage. *183*, 788–799.
28. Swann, N.C., de Hemptinne, C., Miocinovic, S., Qasim, S., Wang, S.S., Ziman, N., Ostrem, J.L., San Luciano, M., Galifianakis, N.B., and Starr, P.A. (2016). Gamma Oscillations in the Hyperkinetic State Detected with Chronic Human Brain Recordings in Parkinson's Disease. J. Neurosci. *36*, 6445–6458.
29. Little, S., Pogosyan, A., Neal, S., Zavala, B., Zrinzo, L., Hariz, M., Foltynie, T., Limousin, P., Ashkan, K., FitzGerald, J., et al. (2013). Adaptive deep brain stimulation in advanced Parkinson disease. Ann. Neurol. *74*, 449–457.
30. Sun, F.T., and Morrell, M.J. (2014). Closed-loop neurostimulation: the clinical experience. Neurotherapeutics *11*, 553–563.
31. Foss, J.M., Apkarian, A.V., and Chialvo, D.R. (2006). Dynamics of pain: fractal dimension of temporal variability of spontaneous pain differentiates between pain States. J. Neurophysiol. *95*, 730–736.
32. Hafner, D., Lillicrap, T., Ba, J., and Norouzi, M. (2020). Dream to Control: Learning Behaviors by Latent Imagination, arXiv, 1912.01603. https://arxiv.org/abs/1912.01603.
33. Yu, T., Finn, C., Xie, A., Dasari, S., Zhang, T., Abbeel, P., and Levine, S. (2018). One-Shot Imitation from Observing Humans via Domain-Adaptive Meta-Learning, arXiv, 1802.01557. https://arxiv.org/abs/1802.01557.
34. Hirata, M., Matsushita, K., Suzuki, T., Yoshida, T., Sato, F., Morris, S., Yanagisawa, T., Goto, T., Kawato, M., and Yoshimine, T. (2011). A fully-implantable wireless system for human brainmachine interfaces using brain surface electrodes: W-herbs. IEICE Trans. Commun. *94*, 2448–2453.
35. Eippert, F., Bingel, U., Schoell, E.D., Yacubian, J., Klinger, R., Lorenz, J., and Büchel, C. (2009). Activation of the opioidergic descending pain control system underlies placebo analgesia. Neuron *63*, 533–543.
36. Bantick, S.J., Wise, R.G., Ploghaus, A., Clare, S., Smith, S.M., and Tracey, I. (2002). Imaging how attention modulates pain in humans using functional MRI. Brain *125*, 310–319.
37. Valet, M., Sprenger, T., Boecker, H., Willoch, F., Rummeny, E., Conrad, B., Erhard, P., and Tolle, T.R. (2004). Distraction modulates connectivity of the cingulo-frontal cortex and the midbrain during pain–an fMRI analysis. Pain *109*, 399–408.
38. Bräscher, A.-K., Becker, S., Hoeppli, M.-E., and Schweinhardt, P. (2016). Different Brain Circuitries Mediating Controllable and Uncontrollable Pain. J. Neurosci. *36*, 5013–5025.

39. Salomons, T.V., Johnstone, T., Backonja, M.-M., Shackman, A.J., and Davidson, R.J. (2007). Individual Differences in the Effects of Perceived Controllability on Pain Perception: Critical Role of the Prefrontal Cortex. J Cogn Neurosci. *19*, 993–1003.

40. Salomons, T.V., Nusslock, R., Detloff, A., Johnstone, T., and Davidson, R.J. (2015). Neural Emotion Regulation Circuitry Underlying Anxiolytic Effects of Perceived Control Over Pain. J Cogn Neurosci. *27*, 222–233.

41. Bingel, U., Lorenz, J., Schoell, E., Weiller, C., and Büchel, C. (2006). Mechanisms of placebo analgesia: rACC recruitment of a subcortical antinociceptive network. Pain *120*, 8–15.

42. Wager, T.D., Rilling, J.K., Smith, E.E., Sokolik, A., Casey, K.L., Davidson, R.J., Kosslyn, S.M., Rose, R.M., and Cohen, J.D. (2004). Placebo-induced changes in FMRI in the anticipation and experience of pain. Science *303*, 1162–1167.

43. Yoshida, W., Seymour, B., Koltzenburg, M., and Dolan, R.J. (2013). Uncertainty increases pain: evidence for a novel mechanism of pain modulation involving the periaqueductal gray. J. Neurosci. *33*, 5638–5646.

44. Taylor, V.A., Chang, L., Rainville, P., and Roy, M. (2017). Learned expectations and uncertainty facilitate pain during classical conditioning. Pain. *158*, 1528–1537.

45. Zhang, S., Mano, H., Ganesh, G., Robbins, T., and Seymour, B. (2016). Dissociable learning processes underlie human pain conditioning. Curr. Biol. *26*, 52–58.

46. Friston, K.J. (2003). Statistical parametric mapping. In Neuroscience databases (Springer).

47. Esteban, O., Blair, R., Markiewicz, C.J., Berleant, S.L., Moodie, C., Ma, F., Isik, A.I., Erramuzpe, A., Goncalves, M., Poldrack, R.A., and Gorgolewski, K.J. (2017). poldracklab/fmriprep: 1.0.0-rc5. https://zenodo.org/record/996169#.XyHJLjpKg2w.

48. Abraham, A., Pedregosa, F., Eickenberg, M., Gervais, P., Mueller, A., Kossaifi, J., Gramfort, A., Thirion, B., and Varoquaux, G. (2014). Machine learning for neuroimaging with scikit-learn. Front. Neuroinform. *8*, 14.

49. Yamashita, O., Sato, M.A., Yoshioka, T., Tong, F., and Kamitani, Y. (2008). Sparse estimation automatically selects voxels relevant for the decoding of fMRI activity patterns. Neuroimage *42*, 1414–1429.

50. Vallat, R. (2018). Pingouin: Statistics in Python. Journal of Open Source Software *3*, 1026.

51. Renard, Y., Lotte, F., Gibert, G., Congedo, M., Maby, E., Delannoy, V., Bertrand, O., and Lecuyer, A. (2010). OpenViBE: An Open-Source Software Platform to Design, Test and Use Brain-Computer Interfaces in Real and Virtual Environments. Presence Teleoperators & Virtual Environments. Presence: teleoperators and virtual environments *19*, 35–53.

52. Cortese, A., Amano, K., Koizumi, A., Kawato, M., and Lau, H. (2016). Multivoxel neurofeedback selectively modulates confidence without changing perceptual performance. Nat. Commun. *7*, 13669.

53. Emmert, K., Kopel, R., Sulzer, J., Brühl, A.B., Berman, B.D., Linden, D.E.J., Horovitz, S.G., Breimhorst, M., Caria, A., Frank, S., et al. (2016). Meta-analysis of real-time fMRI neurofeedback studies using individual participant data: How is brain regulation mediated? Neuroimage *124* (Pt A), 806–812.

54. Koizumi, A., Amano, K., Cortese, A., Shibata, K., Yoshida, W., Seymour, B., Kawato, M., and Lau, H. (2017). Fear reduction without fear through reinforcement of neural activity that bypasses conscious exposure. Nat Hum Behav. *1*, 0006.

55. Nicholson, A.A., Rabellino, D., Densmore, M., Frewen, P.A., Paret, C., Kluetsch, R., Schmahl, C., Théberge, J., Neufeld, R.W.J., McKinnon, M.C., et al. (2017). The neurobiology of emotion regulation in posttraumatic stress disorder: Amygdala downregulation via real-time fMRI neurofeedback. Hum. Brain Mapp. *38*, 541–560.

56. Sherwood, M.S., Parker, J.G., Diller, E.E., Ganapathy, S., Bennett, K.B., Esquivel, C.R., and Nelson, J.T. (2019). Self-directed down-regulation of auditory cortex activity mediated by real-time fMRI neurofeedback augments attentional processes, resting cerebral perfusion, and auditory activation. Neuroimage *195*, 475–489.

57. Sutton, R.S., and Barto, A.G. (2018). Reinforcement learning: An introduction (MIT press).

58. Desikan, R.S., Ségonne, F., Fischl, B., Quinn, B.T., Dickerson, B.C., Blacker, D., Buckner, R.L., Dale, A.M., Maguire, R.P., Hyman, B.T., et al. (2006). An automated labeling system for subdividing the human cerebral cortex on MRI scans into gyral based regions of interest. Neuroimage *31*, 968–980.

59. Ezra, M., Faull, O.K., Jbabdi, S., and Pattinson, K.T. (2015). Connectivity-based segmentation of the periaqueductal gray matter in human with brainstem optimized diffusion MRI. Hum. Brain Mapp. *36*, 3459–3471.

60. Eklund, A., Nichols, T.E., and Knutsson, H. (2016). Cluster failure: Why fMRI inferences for spatial extent have inflated false-positive rates. Proc. Natl. Acad. Sci. USA *113*, 7900–7905.

61. Yarnitsky, D., and Ochoa, J.L. (1990). Studies of heat pain sensation in man: perception thresholds, rate of stimulus rise and reaction time. Pain *40*, 85–91.

62. Fust, J., Lalouni, M., Lundqvist, V.V., Warnberg, E., and Jensen, K.B. (2020). Offset analgesia and onset hyperalgesia with different stimulus ranges. medRxiv, 2020.06.01.20113613.

63. Derbyshire, S.W.G., and Osborn, J. (2009). Offset analgesia is mediated by activation in the region of the periaqueductal grey and rostral ventromedial medulla. Neuroimage *47*, 1002–1006.

64. Maheu, M., Dehaene, S., and Meyniel, F. (2019). Brain signatures of a multiscale process of sequence learning in humans. Elife *8*, e41541.

CellPress
OPEN ACCESS

STAR★METHODS

KEY RESOURCES TABLE

REAGENT or RESOURCE	SOURCE	IDENTIFIER
Software and Algorithms		
MATLAB (2016a)	The MathWorks	https://www.mathworks.com/products/matlab.html; RRID: SCR_001622
SPM12 (6906)	[46]	https://www.fil.ion.ucl.ac.uk/spm/software/spm12; RRID: SCR_007037
fmriprep (0.4.4)	[47]	https://github.com/poldracklab/fmriprep; RRID: SCR_016216
Nilearn (0.6.2)	[48]	https://nilearn.github.io/; RRID: SCR_001362
Sparse Logistic Regression (v1.51)	[49]	https://bicr.atr.jp/:oyamashi/SLRWEB.html; RRID: None
the Decoding Toolbox (v3.98)	[20]	https://sites.google.com/site/tdtdecodingtoolbox/; RRID: SCR_017424
Pingouin (0.3.3)	[50]	https://pingouin-stats.org/; RRID: None
OpenViBe (2.2.0)	[51]	http://openvibe.inria.fr/; RRID: SCR_014156
Deposited code	This study	https://github.com/syzhang/coadapt_repo
Deposited neuroimaging data	This study	https://openneuro.org/datasets/ds002596

RESOURCE AVAILABILITY

Lead contact
Further information and requests for resources/code should be directed to the Lead Contact, Suyi Zhang (suyi.zhang@ndcn.ox.ac.uk).

Materials availability
This study did not generate new unique reagents.

Data and code availability
The MATLAB code for data preprocessing, feature extraction, cross validation, and decoder training has now been uploaded to accompany the manuscript, which can be found on the GitHub repository (https://github.com/syzhang/coadapt_repo). The readme and comments in the code should explain the processing steps in Method Details.

Original data have been deposited to OpenNeuro. All neuroimaging data (functional and de-faced anatomical scans) is available in BIDS format at https://openneuro.org/datasets/ds002596 (OpenNeuro data: https://doi.org/10.18112/openneuro.ds002596.v1.0.1).

EXPERIMENTAL MODEL AND SUBJECT DETAILS

Participants
Experiment 1
19 healthy participants enrolled in a two-day neuroimaging experiment (two females, age 23.5 ± 4.0 years). All subjects gave informed consent prior to participation, had normal or corrected to normal vision, and were free of pain conditions or pain medications. The experiment was approved by the Ethics and Safety committee of the Advanced Telecommunications Research Institute (ATR), Japan (approval number: 16-182). It should be noted that the relatively small sample size here is consistent with previous fMRI-based decoded neurofeedback studies (10-20 participants) [11, 52–56].
Experiment 2
28 healthy participants were assigned respectively to the EEG experimental group (14 female, age 28.8 ± 6.9 years) and the control group (14 female, age 27.1 ± 10.9 years, independent t test between groups $T(27) = 0.661$, $p = 0.511$). All participants gave informed consent prior to the experiment, and were free of pain conditions or pain medications. Ethical approval was granted by the Research Ethics Committee of the Department of Engineering, University of Cambridge.

METHOD DETAILS

Experiment 1: fMRI-based closed-loop control
Experimental protocol
The experiment spanned two days. Each day began with a pain intensity setting procedure outside the scanner, followed by the task. Both days involved 6 sessions with repeated high/low painful stimuli inside the scanner.

Day 1: Decoder construction. Individual participant's functional brain images were recorded during fMRI scanning for decoder training. High and low levels of painful electrical stimuli, determined with the participant's pain threshold obtained before task (see 'Pain calibration procedure' below), were delivered in a sequence of random or pseudo-random trials to elicit two levels of pain. From the participant's perspective, a painful stimulus was delivered at the beginning of each trial when a '+' symbol appeared on screen below a white bulls-eye fixation point. The '+' stayed on for 10 s, then the ' = ' symbol replaced it for 2 s, signaling a brief inter-trial interval (ITI). In 40% trials (12 randomly chosen out of 30 in each session), the '+' stayed on screen for 4 s and the fixation point turned to an orange square signaling upcoming rating, followed by a 0-10 visual analog scale (VAS) that stayed on for 6 s, during which participants were asked to rate how painful the stimulus was by pressing two buttons to move the slider on screen. The 30-trial session was repeated 6 times with a short break in between (180 trials, 72 ratings per subject in total).

Sixteen out of 19 participants used another participant's day 2 trial sequences on day 1, to provide a yoked control, given the plan to directly compare day 1 and day 2 behavioral and brain responses (the initial 3 participants used random sequences). All participants were given the instruction to rest in the scanner and do nothing (see 'Appendix'). Individual-specific, multi-voxel decoder was then trained for automatic classification of pain level experienced, using bilateral insula as region of interest (ROI, see 'Decoder construction' below).

Day 2: Adaptive control. On day 2, the level of pain stimuli delivered on each trial (i.e., the high or low pain stimulator) was controlled by a computer algorithm, whose sole input was the decoded pain probability from the real-time brain response from the previous trial. All subjects were explicitly told that the pain level they received was controlled by the computer, and were aware that modulating their brain activity could therefore influence the computer. Although it could in principle be directly instruct subjects to do enhance MVPA decodability, this creates two difficulties. First, in the absence of any other task, it may be less meaningful to subjects than allowing them to understand the concept of a machine being able to clearly read their pain signals; and second, to make the task incentive compatible, subjects should be free to communicate freely. The instructions are detailed in the Appendix, and were intended to reveal the incentive to enhance pain representations in the brain, but without any explicit instruction on whether or how to do so.

Specifically, after delivering the pain stimulus, a decoder estimated the participants' probability of experiencing high pain (P(Pain)) / low pain by multiplying day 1 decoder weights with the real-time insula BOLD response from their brain images in that trial (realigned and resliced to the reference image from day 1, following Shibata et al. [11], see 'Decoder construction' below). The estimated probability was used to provide the feedback signal with the aim that the computer could learn to deliver less pain to the subject, based on trial-by-trial updating of the decision (action) values associated with triggering each electrical pain stimulator calculated from a basic reinforcement learning algorithm (an 'action' that elicited a low decoded pain signal in the subject was effectively reinforced, see 'Adaptive control' below). An above-chance decoder on day 2 would lead to a greater number of low pain stimuli, which could impair day 1 decoder classification learning because of an unbalanced high/low stimulus frequency in the yoked sequences. However, the actual decoding accuracy and the nature of the reinforcement learning (RL) control function only led to a very modest reduction in high pain stimuli, yielding a sufficient balance of high/low stimuli for classification.

The primary reason for using an adaptive decision function in which the control algorithm learns decision values slowly over time, as opposed to a fixed decision function in which control feedback is fixed based purely on the previous trial, was to maximize the context for communication. That is, the goal of the subject is to teach the machine, and the effectiveness of their ability to communicate is then embedded in the machine memory for future trials, not just the next trial.

Day 1 and 2 were structurally the same apart from the adaptive control process and subject instructions, which allowed approximately yoked conditions permitting investigation of day 1 versus day 2 changes. Across any analysis of effect × day interactions, this sequential comparison necessarily introduces an order confound related to possible non-specific effects of novelty and anxiety to the experiment. Most of these are mitigated by the computational specificity of the analyses, and the within-day contrasts. Notwithstanding this, the effects of interest occur on day 2, when novelty and anxiety effects would be reduced.

Stimulus delivery

Painful electrical stimuli were delivered using two constant current stimulators (Digitimer model DS7A, Welwyn Garden City, Hertfordshire, UK), at two current levels for high/low pain determined using the participant's own threshold. The levels were fixed across sessions but were allowed to differ on day 2 based on the new pain calibration. All stimuli were delivered with a trigger pulse as a train of 50 × 5ms square waves, lasting 500ms (DS7 settings: output scale × 1 mA, pulse duration 200μs). There were no significant differences in the stimulation current levels given between days (paired t test $p = 0.12$ and 0.27 for low and high pain respectively). And there were no significant differences across days for high or low pain levels across individuals (high pain $T(18) = -1.58$, $p = 0.131$, low pain $T(18) = -1.13$, $p = 0.273$), or within sessions (day 1: $F(5,90) = 1.37$, $p = 0.25$, day 2: $F(5,90) = 0.11$, $p = 0.99$, repeated-measure ANOVA pain levels: $F(1,11) = 86.00$, $p<1e-5$), or significant interaction between sessions and days (day: $F(1,11) = 3.173$, $p = 0.103$, session: $F(5,55) = 0.470$, $p = 0.797$). The two stimulators were connected to a switch that allowed current delivery through the same, MRI-compatible concentric ring electrode (10mm diameter). The electrode was taped to the back of the left hand of the participant, its location marked on day 1 as reference for attachment on day 2.

Pain intensity setting procedure (day 1 and 2)

On each day, participants completed an intensity setting procedure at the beginning of the experiment. In the first session, the staircase method was used to evaluate their highest pain limit. Stimuli current were increased at 0.2-0.5mA interval, and participants were asked for verbal feedback of a 0-10 pain rating in person after each stimulation. This procedure was rerun a few times using different starting points and both stimulators. In the second session, 14 trials of randomized painful stimuli were given within the range of lowest perceivable to highest tolerable current level determined in session 1. Subjects rated each stimulus 1 s after receiving it,

on a 0-10 VAS scale on screen using a keyboard (as practice to the rating procedure used in the task). To determine the final current level to use, a Weibull and Sigmoid function were fitted to session 2's stimuli and ratings, and current levels for VAS = 1 and 8 were used for low / high pain stimulus for the experiment respectively. The same procedure was repeated for day 2, and the new fitted current levels were used.

Behavioral data analysis

All statistical tests were conducted two-sided, with Pingouin 0.3.3 in Python 3.

fMRI data acquisition (day 1 and 2)

Neuroimaging data was acquired with a 3T Siemens Prisma scanner with the standard 64 channel phased array head coil. Whole-brain functional images were collected with a single echo EPI sequence (repetition time TR = 2000ms, echo time TE = 26ms, flip angle = 80, field of view = 240mm), 33 contiguous oblique-axial slices (voxel size 3.2 × 3.2 × 4mm) parallel to the AC-PC line were acquired. Whole-brain high resolution T1-weighted structural images (dimension 208 × 256 × 256, voxel size 1 × 1 × 1mm) using standard MPRAGE sequence were also obtained. The choice of voxel size/number was to balance the speed of online decoding and anatomical details, and it was similar to that used in previous real-time fMRI decoded neurofeedback studies that used 3-3.5mm^3 voxels [52, 54, 56]. It should be noted that the current resolution cannot support investigation of PAG sub-region activation.

Decoder construction (day 1)

ROI selection. For decoding, we used BOLD responses in bilateral insula cortex, since this is thought to incorporate sub-regions that have a primary role in the coding of pain and has been shown to provide good intensity decoding accuracy in previous studies [10, 16–19]. Based on a pilot test we conducted prior to the experiment, it also provided the most consistent decoding performance compared to a range of candidate ROIs without reslicing empty voxels during ROI normalization.

Preprocessing. All preprocessing were conducted using SPM12 in MATLAB 2016a. The steps were as followed:

- The first non-dummy (4th) scan of the first session on Day 1 was used as a reference scan.
- Individual subject's structural T1 images were coregistered and segmented to MNI space with SPM12's single subject T1 template.
- The resulting inverse transformation matrix was used to normalize the ROIs in anatomical atlas space to individual subject space.
- The resulting warped ROI masks were then coregistered to the reference scan.
- All subsequent scans (both day 1 and 2) in the task were realigned and resliced to the reference scan using SPM12's realign and reslice functions.
- Temporal signals were extracted from voxels using the processed ROI masks for decoder training (see 'Feature extraction' below for denoising procedures).
- Trained decoder weights were extracted along with voxel coordinates, summarized into a txt file to be used on day 2's decoding sessions.

Feature extraction. Time series were extracted from all voxels within the individual's insula ROI. To account for BOLD delay and to minimize motion contamination, the times series from TR 3-5 (4-10 s) were used from each trial, the first two TRs (0-4 s) immediately following pain stimulus were omitted. For denoising, the 5 TRs following 3 dummy TRs at the beginning of each session were used as baseline, each trial ROI time series were normalized by subtracting session baseline mean and divided by baseline standard deviation, then the mean across the TR 3-5 from all trials were extracted for classifier training.

Decoder training. Mean insula voxel activity as feature and high/low pain delivered as label were aggregated across all trials within participant for decoder training. Binary classification by Sparse Logistic Regression (SLR, version 1.51) with variational parameters approximation was used [49]. This results in a sparse matrix of weights for about 5 percent of all voxels within the given ROI. By multiplying weights with feature/voxel intensity signals, the decoder produces the probability of observing current label given trial features (referred as (P(pain) from here, P(pain) = 1 means highly likely to have received high pain, P(pain) = 0 means unlikely to have received high pain, or highly likely to have received low pain). For training, all day 1 trials were used. To estimate decoder accuracy, all trials were partitioned into 10 equal sets with 9 sets for training and 1 set for testing (10-fold cross-validation) (Table 1).

Adaptive control algorithm (day 2)

To allow automated adaptive control of pain stimulus delivery, we used a simple reinforcement learning algorithm [57] to update the value of high/low pain states trial-by-trial:

$$Q_{t+1}(a) = Q_t(a) + \alpha(-P(pain) - Q_t(a)) \quad \text{(Equation 1)}$$

where t represents trials, Q is the value of given state, a is the actions available for the algorithm (i.e., either giving high or low pain, collectively shown as action set A), α is learning rate fixed at 0.5. P(pain) is the decoder-generated probability of current trial's stimulus being high pain. It's scaled between [−1,1] when used in the updating function. Higher P(pain) would decrease the value of current pain state more and vice versa, while the value of un-chosen state remained unchanged. The algorithm selects which pain level to deliver for the next trial using an ε-greedy action selection rule based on current values:

$$p_{t+1}(a|Q_t) = \begin{cases} random\ action\ a \in A,\ if\ \xi > \varepsilon \\ argmax_{a \in A} Q_t(a), otherwise \end{cases} \quad \text{(Equation 2)}$$

where ε is the explore ratio fixed at 0.4 (i.e., exploring by choosing a random action by either giving high or low pain 40% of the time, exploiting the other times), ξ is a uniform random number drawn within [0,1] at each trial. The noisy exploration allows a sufficient proportion of the alternative electrical stimulator (i.e., pain level) to be delivered, to ensure the next participant who uses current participant's day 2 sequence to have enough trials of both high and low pain for decoder construction. We also set values to be 0 for both states at the beginning of each session.

fMRI data offline analyses

Preprocessing. For offline analysis, functional images were preprocessed using the fmriprep software, a pipeline that performs slicetime correction, motion correction, field unwarping, normalization, field bias correction, and brain extraction using a various set of neuroimaging tools available. The confound files output by fmriprep include the following signals: mean global, mean white matter tissue class, three FSL-DVARS (stdDVARS, non-stdDVARS and voxel-wise stdDVARS), framewise displacement, six FSL-tCompCor, six FSL-aCompCor, and six motion parameters (matrix size 24 × number of volumes). Resulting functional images were smoothed with an 8mm Gaussian kernel in SPM12, except for those in used searchlight analysis.

fMRI GLM model. All event-related fMRI data were analyzed with GLM models constructed using SPM12, estimated for each participant in the first level. Stick functions at pain stimulation onset were convolved with a canonical hemodynamic response function (HRF). We also included rated trials (duration = 10 s, from beginning until ITI) as regressor of no interest, in addition to the 24 columns of confound matrix output by fmriprep. Day 1 and 2 data were included in the same GLM as different sessions with their own intercepts, but first-level contrasts were estimated separately for days.

Whole-brain univariate comparison (Figure 3c). 2 regressors: high/low pain onset (duration = 0).

Frequency learning posterior probability and entropy (Figure 4a). Three regressors at pain onset (duration = 0) with parametric modulators: posterior probability of current stimulus (updated prediction), entropy of previous posterior probability of current stimulus (uncertainty of prediction before updating), actual identity of stimulus (high pain = 1, low pain = −1). All parametric modulators mean centered within session, SPM orthogonalisation for these 3 regressors were turned off. Posterior probability and entropy uncertainty were not highly correlated (n = 19, mean correlation r = 0.0663, std = 0.119, one sample t test against mean 0: t = 2.43, p = 0.0258).

Correction for multiple comparison. We use whole brain correction or ROI based correction based on *a priori* hypotheses as appropriate, and the details appear in Table 2. For ROI analyses, we used anatomical binary masks generated using the Harvard-Oxford Atlas [58] for clearer labeling (freely available with the FSL software, https://fsl.fmrib.ox.ac.uk/fsl/fslwiki/Atlases), and PAG probabilistic atlas [59] for small volume correction. We used the frontal medial cortex mask as approximation for VMPFC. We also used the pgACC peak identified in our previous study of active relief learning [14] for the 8mm spherical ROI mask (sphere peak used: [6, 40, 12]), given there are no specific ROI mask from anatomical atlases for the region. We reported all results with p<0.05 (FWE cluster-level corrected, using a p<0.001 cluster-forming threshold [60]), with the exception of searchlight analysis results (MFG/DLPFC SVC had p = 0.06, see Table 2).

ROI analysis. For testing ROI significance in experimental conditions, beta estimates were extracted from activation ROIs (see text for mask details). Beta values plotted were the average of all voxels within ROI masks, with statistics showing subject-level SEM (Figure S2). All t tests performed were two-tailed. Statistical maps overlaid on subject-averaged anatomical scans using Nilearn. For testing statistical significance in GLM analyses, we used voxel-wise correction for multiple comparisons within the ROIs: the insula (required by the task paradigm itself, and the pgACC and PAG given their proposed role in cognitive control [14, 21]). Different ROIs are being tested separately for multiple comparison with relatively lenient correction thresholds, however, these clusters came from separate GLM analyses designed to test for different effects of the experiment.

Decoder comparison. Decoders were constructed using day 2 data with the same procedure as day 1 (Figure 3). This was done to determine whether the decoding performance of insula ROI remained the same, or whether any learning-induced changes might have changed the decoder properties. Whole-brain searchlight analysis was conducted using the Decoding Toolbox. The toolbox can conduct multivariate decoding analyses at combined trial types within fMRI runs, by extracting features from beta images of relevant regressors in the first level GLM analysis output by SPM. This could lead to higher classification accuracy and lower computation time, comparing to single trial decoding.

A searchlight analysis was carried out within a 10mm radius sphere for the whole brain, with high/low pain categories as unsmoothed beta images from each run for individual participant. TDT toolbox produced a decoding accuracy map for each voxel using a leave-one-run-out cross validation scheme, which can be interpreted as the local information content of each voxel [1]. The day 1 and 2 accuracy maps from each individual were then smoothed with a Gaussian kernel of 4mm, and entered into a standard SPM second level paired t test as in the GLM analysis above. The resulting T map indicates the changes in decodable information used for pain level decoding across days.

Experiment 2: EEG-based closed-loop control

The adaptive control paradigm itself was very similar to the first experiment, but incorporated four key differences. First, we used EEG instead of fMRI for neural recording, since this allows much more efficient data collection in terms of time and cost, as well as easier clinical translatability. Second, unbeknownst to the subjects, we used random feedback (i.e., sham EEG decoding) so that the engagement of endogenous modulation would be due purely to the subjects' active attempts to engage (e.g., enhance communication) with the machine, and not as a result of any neurofeedback conditioning by successful relief attainment (see discussion [54],). Third, we employed a control condition (i.e., a separate group of participants) that did not involve any brain recording or adaptive

control, to control for potential order confounds in the fMRI experiment (at least in terms of the behavioral effects). Fourth, the pain stimulus was delivered to the lower back because this is the most common site of clinical chronic pain and hence a target for future therapeutic closed-loop systems.

Experimental group

Experimental protocol. In a similar manner to the first experiment, the experimental participants in the adaptive control group were given the instructions that their pain stimulation was determined adaptively by their real-time EEG brain responses, and they understood that they could use different cognitive strategies to better communicate their pain to the machine. The control task was designed to administer the same number of pain stimuli but in a completely different context to the adaptive control task. Instead, control participants were asked to passively listen to a podcast (an audio book), and simply needed to rate pain intensity intermittently (Figure 1b, d). This provided a neutral cognitive condition that allowed us to control for any non-specific changes to pain related to habituation or sensitization in the context of a laboratory experiment that engaged a baseline level of attention. Both groups received a high/low pain stimulus at around 50% chance level. As in the fMRI experiment, there were no significant differences in the overall average pain stimulation ratings between groups (repeated-measure ANOVA pain level × group interaction p>0.5). Without the need for fMRI volume acquisition, variable ITI was used (trial time mean = 8.7 s, std = 0.49 s), otherwise trial structure remained the same. Each participant completed 8 sessions of 30-trial experiment, while also completing the thermal temporal contrast enhancement test before and after these sessions.

EEG data acquisition. EEG data were collected using an 8-channel system (g.Nautilus, g.tec GmbH, Austria) with accompanying gel-based electrodes placed on a cap according to the international 10-20 system (Fz, Cz, Pz, C3, C4, T3, T4, and a surface electrode was placed 10mm below the left eye to monitor eye movements), with a sampling rate of 250Hz. The nose was used as reference, and electrode impedance were kept under 30$k\Omega$. EEG data were streamed and saved using OpenViBe. Despite the set-up, the design of this experiment involved giving random feedback to the subjects, to remove the chance that a high number of positive outcomes (i.e., low pain) would have a reinforcing feedback effect. In another manuscript we aim to present a full EEG-based adaptive control framework based on decoded EEG, but we would note here that it is clear that the decoding accuracy based on EEG is substantially lower than fMRI, and so a robust and effective closed-loop system is more difficult to establish.

Control group

Experimental protocol. Control group participants did not have EEG recordings. They were asked to listen to an audio podcast of their choice (from BBC Sounds website, contents include stories and discussions) while receiving electrical stimulation and to complete the same pain rating procedures during the stimulation sessions as experimental group.

Temporal contrast enhancement paradigm

Participants from both experimental and control groups completed a thermal temporal contrast enhancement paradigm, before and after the main experimental session. Temporal contrast enhancement refers to the 'change hypersensitivity' typically seen in pain ratings: when a tonic pain stimulus is slightly increased or decreased, there is an unexpectedly large (compared to steady temperature state ratings) increase or decrease in ratings. This is sometimes called 'onset hyperalgesia' and 'offset analgesia' respectively [25, 27, 61, 62],), and although it may have actually been driven by multiple mechanisms, the dominant mechanisms is thought to be facilitation and inhibition with the descending endogenous control system. Heat pain stimulation were delivered with the contact heat-evoked potential stimulator (CHEPS, Medoc Pathway, Israel) to the skin on the participant's lower back. Participants rated their pain continuously on a 0-10 scale during the 3 stages of temperature: 45°C (T1, 7 s) - 46°C (T2, 7 s) - 45°C (T3, 7 s) (35°C baseline, ramp rate 10°C/s, ITI = 7 s, 5 trials in total) [63].

To quantify endogenous modulation during the task results, we z-score normalized continuous ratings within individual (excluding T1 ratings from 0-6 s, since they did not contribute to magnitude calculation and could add to rating variance), resampled at 1 s, and averaged across participants. The endogenous modulation magnitude is defined as $T2_{max} - T3_{min}$ using individually processed normalized pain ratings, before comparing across groups [26].

Electrical stimulus delivery

Identical constant current stimulators were used to deliver painful electrical stimuli to participants, with similar pain calibration procedures (see 'Stimulus delivery' and 'Pain calibration procedure' above). A pair of disposable surface electrodes (diameter 20 × 25mm, electrode distance 1cm) were used to deliver stimulation to participant's lower back on the contralateral side that received thermal stimulation. Comparing to the ring electrode, surface electrodes increased the discriminability of pain levels by recruiting a larger number of fibers (due to electrode differences the electrical current levels were not directly comparable between experiments). There were no significant differences in stimuli levels between experimental and control groups (high pain: T(27) = −0.484, p = 0.630, low pain: T(27) = −1.65, p = 0.104).

Frequency learning model

The frequency learning model M assumes a participant estimates the posterior distribution of a given stimuli θ from a previously observed sequence of two possible stimuli $y_{1:t}$ (i.e., high or low pain) using Bayesian updating [23, 24].

$$p(\theta|y_{1:t}, M) \propto p(y_{1:t}|\theta, M)p(\theta, M) \quad \text{(Equation 3)}$$

Given the experimental design, participants are assumed to have uninformative prior over the two stimuli at the beginning of each session, which can be represented by a Beta distribution with parameters [1, 1]. Since the product of two Beta distributions results

in a Beta distribution, the posterior distribution depends only on the frequency of the high and low stimuli N_h, N_l, which has an analytical solution. The posterior mean of the predicted high pain distribution is:

$$p(h|N_h, N_l) = \frac{N_h + 1}{N_h + N_l + 2} \quad \text{(Equation 4)}$$

and $P(l|N_h,N_l) = 1 - p(h|N_h,N_l)$ given the reciprocal relationship between high/low pain stimuli.

It is possible that the number of trials for frequency memory is limited due to memory constraints. This can be modeled by introducing a forgetting 'leaky factor' ω to exponentially decay the number of previous observations, where trials closer to the present are weighted higher [23, 64]. The weighted number of observations was calculated as:

$$N_h^{\omega} = \sum_{t=1}^{n} u_{n-t}^{-\exp\left(\frac{-t}{\omega}\right)} \quad \text{(Equation 5)}$$

where $u_{1:t}$ is the sequence of trials encoded with 1 s and 0 s that represent high and low pain respectively.

Participants were assumed to accumulate stimulus evidence over the entire session (30 trials), where we assumed either perfect (no leaky factor) or imperfect memory retention (with leaky factor ω). We assumed subjects reset their prior expectation at the beginning of each session because there were natural breaks between fMRI sessions with blank screens, during which we asked them for brief verbal feedback on their pain levels and performance estimation after each session. Participants in both EEG groups were explicitly told that sessions were not related to each other.

The uncertainty/surprise of current stimulus *h/l* at trial *t* can be estimated as the entropy *H* of the posterior mean before updating from trial $t-1$:

$$H(P(h_t)) = -log_2(P(h_{t-1})) \quad \text{(Equation 6)}$$

To determine any learning effects on subjective ratings, we followed the method in Woo, Schmidt et al. [10] to use subjective rating residuals for correlation analysis with learning model predictors. We regressed subjective ratings with a matrix of high/low pain stimulus identities (high = 1, low = −1), and session numbers for each individual to obtain rating residuals. The fluctuation of the resulting residuals can be interpreted as modulatory effects on pain beyond the level of nociceptive inputs.

For model fitting, a grid search was run with different leaky integration ω (1-29, or no leak) to produce different sets of model predictors (posterior probability and entropy). For each individual, the regression coefficient β_0 and β_1 were estimated using linear regression model [64]:

$$y_t = \beta_0 + \beta_1 * predictor(\omega) \quad \text{(Equation 7)}$$

where y_t is the rating residuals. The model evidence can be estimated using the Bayesian information criterion (BIC), calculated as followed:

$$BIC = n \cdot \log\hat{\sigma}2 + \kappa \cdot \log n \quad \text{(Equation 8)}$$

$$\hat{\sigma}2 = \min \frac{1}{n} \sum^{n} (y_t - \hat{y}_{t,\omega})^2 \quad \text{(Equation 9)}$$

where n is the number of observations/trials, κ the number of parameters (no leak: 2 (β_0,β_1), leak: 3 (β_0,β_1,ω)), and σ^2 is the mean squared error from regression. Using the grid search, the model with overall lowest BIC (or fitting error) averaged across participants were considered to be the winning model with the best set of parameters (Figure S4).

fMRI experiment participant instructions

Day 1 (Decoder construction). Please rest in the scanner. We are looking at your brain's response to different levels of pain. You don't have to do anything.

Day 2 (Adaptive control). You don't need to do anything in this task. The computer is trying to work out if you feel pain or not, by looking at your brain activity. If it thinks you felt pain, it will try and change the pain stimulation to stop you from having pain. If it thinks you did not feel much pain, it will try not to change anything. However, it cannot do this very reliably, as reading the brain activity is difficult, so it may often make mistakes.

During your first scan, we gave a random sequence of pain stimuli - some high, and some low. Using this data, we have trained a computer program to tell how much pain you were feeling during each shock, based on your brain activity. It is good, but not perfect - it gets it right about 80% of the time.

In today's scan, the computer program can influence the pain level you get. If it thinks you felt a lot of pain, it will influence the pain machine to give you less pain in the future. If it thinks you did not feel much pain, it will try to influence the pain machine to continue to give you little pain. In other words, it is trying to help you get less pain! This is a difficult job for the computer program, because it is not perfect at reading your brain activity as soon as it is active (i.e., within a few seconds).

It is up to you what you do in the task. You can do nothing, and hope that the system works well, and the computer learns to reduce the pain. Or you can try to influence the computer using your thoughts, in any way that you like.

CellPress
OPEN ACCESS

Post-training survey (Day 2)

- Do you think the machine was successful in reading your pain and trying to reduce it?
- Did you try to influence the computer by doing or thinking anything?
- If so, what did you do/think?
- And if so, do you think you were successfully able to influence it?
- Any other comments or feedback?

Current Biology, Volume *30*

Supplemental Information

Pain Control by Co-adaptive Learning in a Brain-Machine Interface

Suyi Zhang, Wako Yoshida, Hiroaki Mano, Takufumi Yanagisawa, Flavia Mancini, Kazuhisa Shibata, Mitsuo Kawato, and Ben Seymour

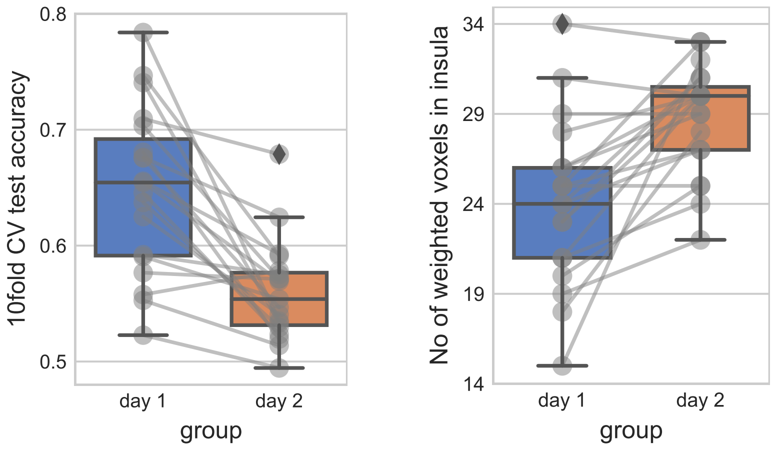

A B

Figure S1: **Decreased pain information in the insula. Related to Figure 3.**

(**A**) The 10 fold cross-validated test accuracy with bilateral insula ROI was 64.8% for day 1 and 56.0% for day 2 (Wilcoxon signed-rank test Z(18)=3.69, p<0.001).
(**B**) The decoder trained with day 2 data identified significantly more voxels as contributing to decoding performance compared to day 1 (day 1: 24.1±1.1 voxels, day 2: 28.7±0.7 voxels, signed-rank test Z(18)=-3.21, p=0.001). The sparse logistic regression method we used prunes unnecessary features/voxels when building the classifier [51]. There were no significant differences in the locations (i.e. average of x, y, or z coordinates) of these weighted voxels within individuals. Therefore both the searchlight and standard ROI decoder results suggest the insula cortex displays a disrupted pattern of information encoding for pain intensity on day 2.

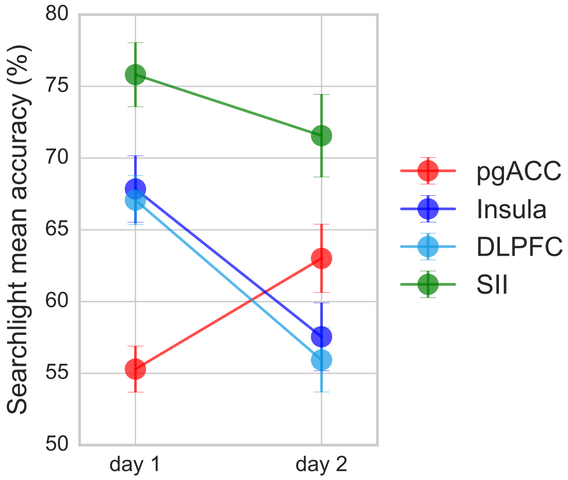

Figure S2: **Increased pain information in the pgACC. Related to Figure 3.**

Comparison between the mean searchlight accuracy map of pgACC and insula clusters (masks extracted from clusters shown in figures below, at $p<0.005$ unc.) and right SII (mask from accuracy>75% on day 1, k=50 voxels). The SII is known to robustly encode pain patterns and so could serve as an independent marker of changes, and its accuracy did not vary significantly across days (averaged within the ROI mask from day 1, k=50, day 1: 75.813 ± 2.234, day 2: 71.563 ± 2.880, paired t-test $T(18)=1.344$, $p=0.196$). A day×location interaction showed a significant regional difference between pgACC and SII ($F(1,18)=12.1$, $p=0.0027$), although not significantly so between insula and SII ($F(1,18)=3.10$, $p=0.095$).

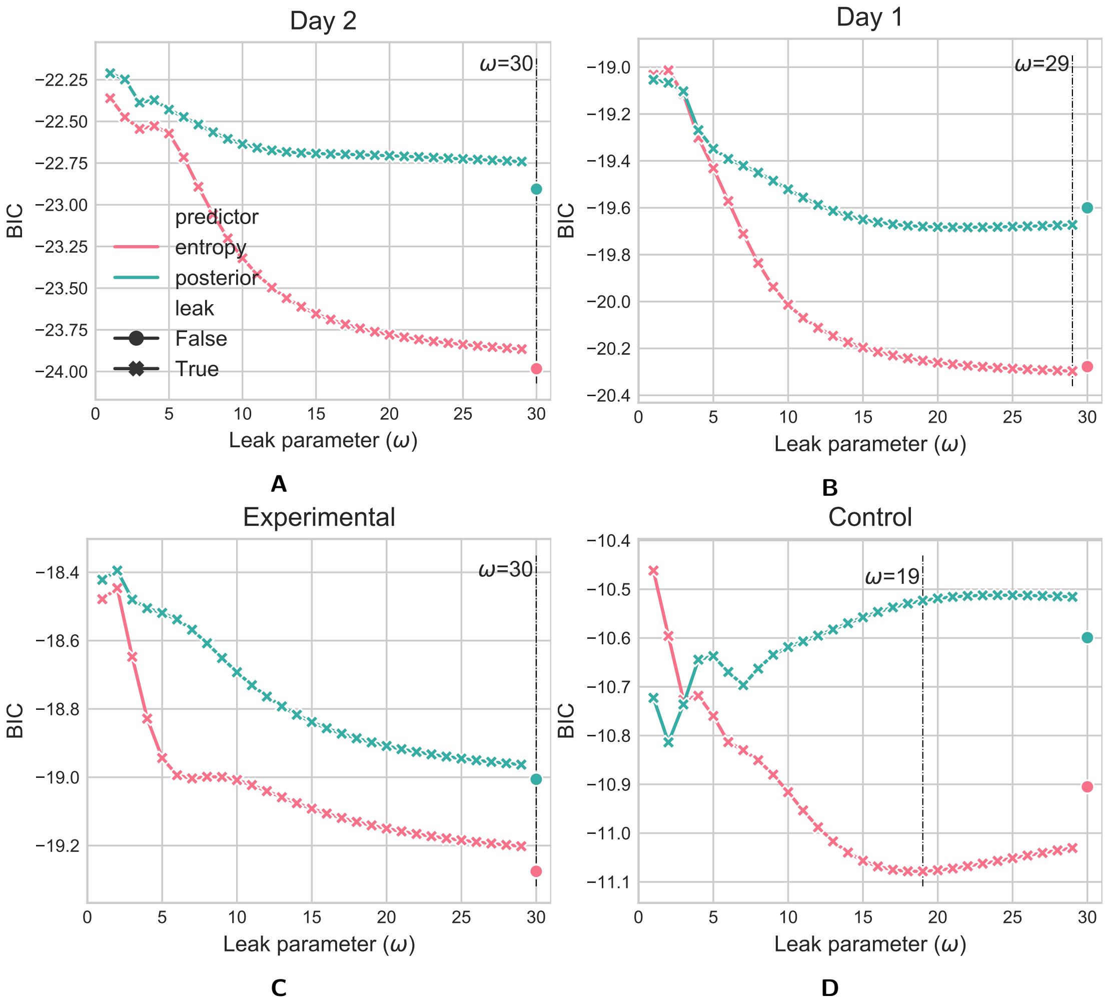

Figure S3: **Uncertainty model fitting and comparison. Related to Figure 4.**

(**A-B**) fMRI experiment: Model comparison showing entropy (formally representing uncertainty regarding upcoming stimulus level) from frequency learning model fitted pain rating residuals best (BIC the lower the better). Model with no leaky integration fitted day 2 data best (i.e. model assumed perfect tracking of trial frequency, day 1 BIC_{min}=-20.30, best-fit leaky parameter=29, day 2 BIC_{min}=-23.98, best-fit parameter: no leak). This suggests the model assumed perfect tracking of high/low pain frequency within a session of 30 trials on day 2, instead of exponentially weighted more recent trials on day 1, although the difference is close to negligible.
(**C-D**) EEG experiment: Model comparison showing entropy with no leak fitted pain rating residuals in the experimental group best (BIC_{min}=-19.28, best-fit parameter: no leak), however for the control group, the best-fit model included an integration parameter ($\omega = 19$, BIC (BIC_{min}=-11.09) that discounts trials frequency earlier in the session.

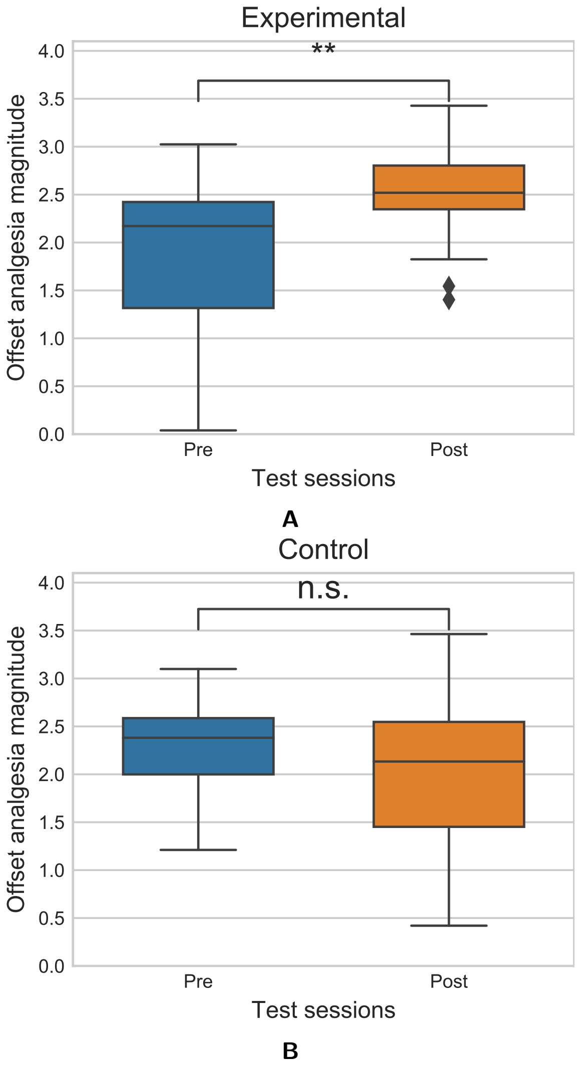

Figure S4: **Adaptive control increased temporal contrast enhancement. Related to Figure 5.**

The magnitude of temporal contrast enhancement before and after the task (pre/post) in the experimental (**A**) and control (**B**) groups. In the pre- and post- experimental temporal contrast enhancement task, subjects experienced a contact thermal pain stimulus that rose from a warm baseline to 45°C for 7 secs (T1), then to 46°C for 7 secs (T2), and then back to 45°C for 7 seconds (T3), and rated pain using a continuous numerical rating scale. Temporal contrast enhancement modulation magnitude is defined as pain ratings $T2_{max} - T3_{min}$.

Subject ID	Do you think the machine could read your pain and thus succeeded in reducing pain?	Did you tried to interact with the machine to exert some influence on it?	Actual strategies used (summarised)
1	Yes	Yes	Sometimes tried to prepare for the stimulus, sometimes simply wished for lower pain.
2	Yes	Yes	Concentrated on pain, several active strategies to change pain in the mind, including exaggerating response (i.e. make low pain lower, high pain higher)
3	Yes	Yes	No clear report of any cognitive strategy, generally passive
4	Yes	Yes	Attended to the pain, explicitly commented on intensity within his mind.
5	Yes	Yes	Thought about the pain, mainly wanting it not to hurt
6	Yes	Yes	Sometimes attended to the pain, including explicitly commented on intensity within his mind.
7	Yes	Yes	Concentrated on pain, sometimes trying to exaggerate the response
8	No	Yes	Mainly tried to relax
9	Yes	Yes	Concentrated on pain, some exaggeration (especially for high pain), related to pain to past memories.
10	Yes	Yes	Range of things: sometimes passive, sometimes attending to, exaggerating pain.
11	Yes	Yes	Sometimes passive, sometimes exaggerating pain
12	Yes	Yes	Attended to pain, monitored frequency, sometimes counting.
13	Yes	Yes	Generally passive
14	Yes	Yes	Focused on anticipating pain, sometimes tried to relate to pain memories
15	Yes	Yes	Tried to anticipate intensities, monitored frequency, often counting
16	Yes	Yes	Generally passive
17	Yes	Yes	Sometimes actively exaggerating pain, other times passive with mind wandering
18	Yes & No	Yes	Often exaggerated pain, other times more passive
19	Yes	Yes	Mainly focusing on pain, trying to decipher intensity clearly.

Table S1: **fMRI experiment post-training survey summary. Related to STAR Methods.**
